# Microbial and Geochemical Dynamics of an Aquifer Stimulated for Microbial Induced Calcite Precipitation (MICP)

**DOI:** 10.3389/fmicb.2020.01327

**Published:** 2020-06-16

**Authors:** J. A. Ohan, S. Saneiyan, J. Lee, Andrew W. Bartlow, D. Ntarlagiannis, S. E. Burns, Frederick S. Colwell

**Affiliations:** ^1^Department of Microbiology, Oregon State University, Corvallis, OR, United States; ^2^Bioscience Division, Los Alamos National Laboratory, Los Alamos, NM, United States; ^3^Department of Earth & Environmental Sciences, Rutgers University, Newark, NJ, United States; ^4^College of Engineering, Georgia Institute of Technology, Atlanta, GA, United States; ^5^College of Earth, Ocean, and Atmospheric Sciences, Oregon State University, Corvallis, OR, United States

**Keywords:** microbial induced calcite precipitation (MICP), biomineralization, biocementation, biostimulation, soil stabilization

## Abstract

Microbially induced calcite precipitation (MICP) is an alternative to existing soil stabilization techniques for construction and erosion. As with any biologically induced process in soils or aquifers, it is important to track changes in the microbial communities that occur as a result of the treatment. Our research assessed how native microbial communities developed in response to injections of reactants (dilute molasses as a carbon source; urea as a source of nitrogen and alkalinity) that promoted MICP in a shallow aquifer. Microbial community composition (16S rRNA gene) and ureolytic potential (*ureC* gene copy numbers) were also measured in groundwater and artificial sediment. Aquifer geochemistry showed evidence of sulfate reduction, nitrification, denitrification, ureolysis, and iron reduction during the treatment. The observed changes in geochemistry corresponded to microbial community succession in the groundwater and this matched parallel geophysical and mineralogical evidence of calcite precipitation in the aquifer. We detected an increase in the number of *ureC* genes in the microbial communities at the end of the injection period, suggesting an increase in the abundance of microbes possessing this gene as needed to hydrolyze urea and stimulate MICP. We identify geochemical and biological markers that highlight the microbial community response that can be used along with geophysical and geotechnical evidence to assess progress of MICP.

## Introduction

Microbially induced calcite precipitation (MICP), the process by which calcium carbonate is precipitated as a result of microbial activity, has been proposed for multiple applications including groundwater remediation, erosion control, stabilization of soils, and sealing of fractures in geological material (Colwell et al., 2005; van Paassen, 2011; DeJong et al., 2013; Phillips et al., 2013). The advantages of MICP include avoiding soil compaction, reducing toxic additives to soils, lowering costs compared to traditional engineered approaches, and taking advantage of the broad distribution of microorganisms in Earth systems (Martiny et al., 2006). The method is gaining favor in a range of settings and represents one of the many bio-mediated or bio-inspired technologies contemplated for geotechnical applications (DeJong and Kavazanjian, 2019).

As with any relatively new engineering strategy, initial lab studies validate the general process and consider how it might be designed to address applied needs such as those identified above. Laboratory scale investigations have demonstrated ways to control MICP, and further scrutiny of the method is occurring in pilot and field-scale tests to determine the conditions under which it can be successfully used and to evaluate the factors that limit its use. Although previous studies have reported properties such as the strength of calcite formed by microbes in the treated soils (Montoya and DeJong, 2015) and changes in geophysical properties caused by MICP (van Paassen et al., 2010a, b; Saneiyan et al., 2019) it is equally important to look for signs in the subsurface medium that indicate the presence and activity of key microbes.

Previous work that examined the role of microbes during MICP has often aimed at detecting the specific cells or functionality needed to accelerate calcite precipitation. Most often the process is implemented in the field by addition of urea to the aquifer to encourage urea hydrolysis by native microbes which is followed by formation of bicarbonate ion as shown in the following reaction (as reported by Fujita et al., 2008)

HNCONH2(urea)2+3HO2->2NH+4-HCO+3-OH-

Microbially induced calcite precipitation results from the increased concentration of bicarbonate in the presence of dissolved calcium ions (abundant in many groundwaters) and the subsequent precipitation of calcium carbonate minerals on aquifer or soil solids. This biologically catalyzed reaction is distinct from abiotic calcite precipitation which occurs much more slowly in many soil and aquifer environments (Reeder, 1983).

Because of these reactions, during MICP field studies, investigators quantify the *ure*C subunit of the microbial gene for urease, an enzyme used by microbes to hydrolyze amendments of urea and thereby alter the pH and alkalinity of the groundwater to enhance calcite precipitation (Fujita et al., 2008). As an example, Fujita et al. (2008) observed a 170-fold increase in *ure*C gene copy numbers above the levels measured before starting the amendments. In another field study, potential urea hydrolysis rates increased in dominant microbes during and following treatment (Colwell et al., 2005). Both of these investigations focused on an explicit microbial function that is crucial for stimulating calcite precipitation. In addition to studying the specific process for urea hydrolysis, it is important to record the evolving structure and function of the broader microbial community because this can lend support to evaluations of successful use of the strategy.

Investigations of how subsurface or aquifer microbial communities change under dynamic chemical or physical conditions (both natural and engineered) are key to many previous field studies. Understanding this biological component, and the associated processes influenced by microbes, is a research need in subsurface science (National Research Council, 2000). Detection of microbial community members responsible for a desired, engineered outcome along with the functional capabilities that the cells possess can be the primary means of interpreting the outcome of a field study (Anderson et al., 2003; Yabusaki et al., 2007; van Nostrand et al., 2011) and can also be used as a criterion for accepting biostimulation as a course of action (Conrad et al., 2010). More generally, assessments of complex and diverse microbial assemblages in aquifers subject to change provide new ways to interpret these biogeochemically dynamic ecosystems (Stegen et al., 2016; Küsel et al., 2016).

Our objective was to measure changes in key microbiological and geochemical properties in an aquifer over the course of an experimental stimulation of microbial induced calcite precipitation. This study integrates biogeochemical measurements with concurrent geophysical and mineralogical measurements made during the same field experiment that show evidence for successful MICP (Saneiyan et al., 2019). Data regarding these microbiological processes can be used in conjunction with other analyses such as those derived from geophysics and geotechnical engineering to help validate MICP in the field.

## Materials and Methods

### Field Site and Hydrogeology

**FIGURE 1:**
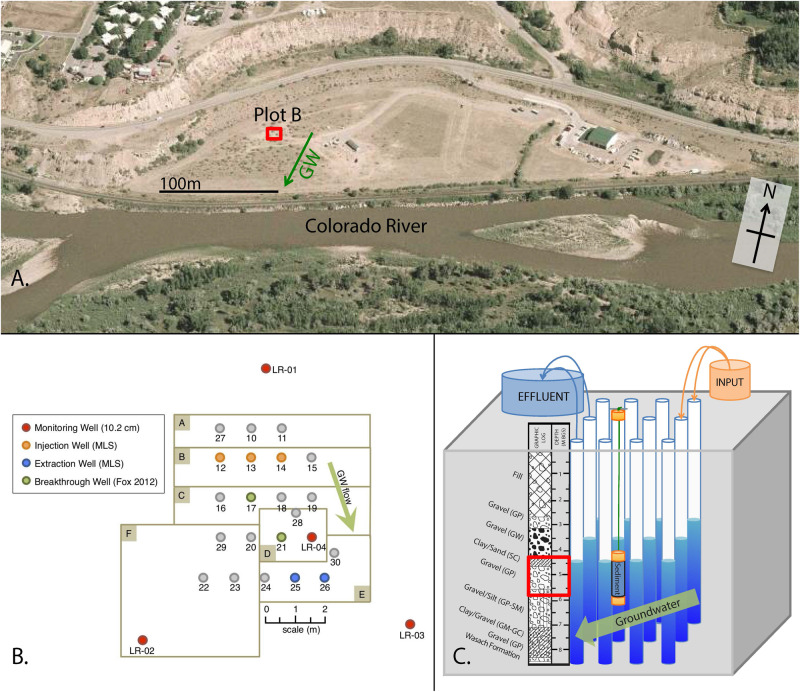
Rifle site location, layout, and injection scheme. **(A)** Aerial photo of Colorado River floodplain showing the Rifle site and Plot B where our study occurred (image from U.S. Department of Energy, 2018), **(B)** Plan view map of Plot B showing well locations (modified from Fox et al., 2012) and **(C)** Oblique view of Plot B showing relative location of injection wells, monitoring wells, and generalized lithology (depth in m below land surface) as determined in monitoring well LR-04. The red box indicates the depth from which all groundwater was sampled in this study (i.e., the depth constrained by the multi-level sampling system).

The field research was conducted at the Old Rifle processing site (Figure 1A), a Uranium Mill Tailings Radiation Control Act (UMTRCA) Title I site where uranium and vanadium ore were processed and disposed of from 1924 to 1958. From 1992 to 1996, contaminated soil was removed from the site and replaced with fill and a relatively impermeable clay cap (Chang, 2005). The site, near the Colorado River, hosts an unconfined alluvial suboxic aquifer underlain by the Wasatch formation which acts as a low permeability aquitard. Groundwater generally flows through the site toward the Colorado River to the south, with recharge inflow from the north from rainfall/snow melt, and seasonally from the Colorado River (U.S. Department of Energy, 1990, 1999; Yabusaki et al., 2017). Prior to our study, organic carbon had not been injected into the subsurface at the specific location of our research (KH Williams [Rifle Site manager], personal communication).

Our study was conducted at a previously drilled well field at the Rifle site (Figure 1B; identified as “Plot B” in Fox et al. (2012) in September 2016 and details of the co-occurring geophysical investigation of MICP are reported elsewhere (Saneiyan et al., 2019). The 16-day period over which our study occurred was consistent with the length of time used in past biostimulation studies conducted in the aquifer at the Rifle Site (Anderson et al., 2003; Smith et al., 2012). Given the southward groundwater flow direction reported by Fox et al. (2012) and others; and not known to reverse direction during the late summer when our study was conducted (Yabusaki et al., 2017) we partitioned the well zones in the projected treatment area to anticipate the injectate delivery (Figure 1B). Groundwater samples for our study were acquired from pre-existing wells with sample ports 4–6 meters below ground surface (Figure 1C) and these samples provided an indication of successive changes occurring in the aquifer during MICP. Fox et al. (2012) calculated the flow velocities to vary between 0.16 and 1.1 m/day depending on vertical location in the aquifer. At the depth in well LR-04 where many of our samples were acquired Fox et al. (2012) reported a flow velocity of 0.63 m/day in an adjacent well. Aquifer recharge is estimated to be 20,000 L/day (Saneiyan et al., 2019). The aquifer at the Rifle site is generally anoxic and has high alkalinity (ca. 10 meq/L) (Yabusaki et al., 2007).

### Injections of Tracers and Compounds to Stimulate MICP

Groundwater was pumped from upgradient wells to minimize and distribute recharge effects, and then stored in a 500-gallon water bladder prior to re-injection with amendments. Subsequently, molasses (#677, Malt Products Corp, Rochelle Park, NJ, United States), urea (46-0-0 NPK fertilizer; Silt, CO, United States), and/or KBr (Sigma-Aldrich) were added and mixed in a surface storage tank to achieve concentrations of 0.13 g/L, 8.325 mM, and 10.15 μM, respectively. KBr was used as a conservative tracer to track injectate delivery. We injected these solutions into wells 12, 13 and 14 (Figure 1B) at a combined rate of 3–4 L/min. Supplementary Table S1 provides details on the schedule and specific volumes of injected fluids and specific additives during the 15-day injection period. While injecting, we pumped water from downgradient wells 25 and 26 at a combined rate of 200–750 mL/min to encourage delivery of amendments. After eight days, the absence of tracer breakthrough at downgradient wells indicated that injectate concentrations were likely insufficient and may have become diluted due to dispersion in the aquifer. At that point, we increased injection events and surface tank concentrations of injectates to 1.11 g/L, 64.75 mM, and 4270 μM for molasses, urea and KBr, respectively, to distribute these compounds more effectively through the aquifer and to more closely match biostimulation studies previously conducted at the Rifle site (Anderson et al., 2003; Smith et al., 2012). We based our overall plan on prior MICP field experience (cf., Colwell et al., 2005; Fujita et al., 2008) and accordingly calculated the approximate conditions for molasses and urea amendments needed to encourage MICP. In planning, we decided not to conduct a negative control experiment (i.e., circulate the aquifer without amendments added) at a duplicate nearby site because the cost and effort to do so would have been excessive.

### Groundwater Geochemistry

We collected groundwater at regular intervals from wells at plot B to preserve for geochemical analysis. Geochemistry of artificial sediments was not performed. To sample, groundwater from 5 m below ground surface (Supplementary Table S2) was purged from sampling lines in the wells and then pumped to fill sterile 50 mL centrifuge tubes, and refrigerated (4–6°C) for chemical analysis as listed below within a day of collection. For anion analysis by ion chromatography (ICS-1000; Dionex), 1.5 mL was aliquoted from the 50 mL samples, filtered with 0.2-um-pore-size PTFE (Teflon) syringe filters (Millipore - Sterivex) and refrigerated (4–6°C). Anion peak values (i.e., bromide, sulfate, nitrate, nitrite) were extracted using Chromeleon software (ThermoFisher Scientific). Using colorimetric test kits and a V-2000 photometer (both from CHEMetrics, Midland, VA, United States), we also measured ammonia, nitrite, and ferrous iron concentrations to evaluate key water chemistry parameters linked with changes in microbial activity associated with MICP. Limits of detection for vacu-vial measurements were as follows: ferrous iron (0 – 84 μM), ammonia (0 – 388 μM), nitrite (0 – 22 μM). Measurements “out of range” were diluted 25-fold to obtain an accurate reading. All samples were not replicated; however, some replication on an intermittent basis verified that measurements were accurate. Specific conductance (a possible indicator of increased ionic strength in groundwater that may occur during MICP due to ion exchange on aquifer solids or presence of bromide tracer) and pH were measured using calibrated probes (Orion 4 Star Plus; Thermo Scientific).

We calculated standard scores (*Z*-scores) as: *z*_i_ = (*x*_i_ – *x*)/s, where *z*_i_ = sample standard score i; *x*_i_ = sample value i; *x* = mean; *s* = standard deviation (Güler et al., 2002). These scores were calculated to equally weight each parameter (geochemical values, i.e., μM) so they could be compared directly to each other. We plotted the *Z*-scores using Microsoft Excel v.14.7.7 to identify general trends in the site’s groundwater geochemistry (Supplementary Figure S1). By correlating geochemical response patterns with the injection scheme, we sectioned the study into “early,” “mid,” and “late” stages, and constructed a conceptual geochemical model.

### Artificial Sediment Columns

Comprehensive analyses of subsurface microbial communities account for both free living (i.e., unattached) and attached microbes because these two populations are known to differ (Lehman, 2007). Our only means of sampling the subsurface at the field site was by pumping groundwater (collecting cores was not possible), so we designed artificial sediment cores that contacted groundwater and allowed microbial colonization either under static or advective flow conditions (Supplementary Figure S2). Conceivably, both static and flowing conditions might occur in an aquifer and accounting for both would allow a more comprehensive assessment of the attached microbes present. For colonization under static conditions, we aseptically assembled artificial sediment columns (hereafter referred to as “sediments”) and then incubated them 5 m below ground surface in wells LR-01, LR-02, and LR-04 (see Figure 1C) in the aquifer. For colonization under advective flow, groundwater pumped from well LR-04 was continuously recirculated through columns kept at the surface in an insulated cooler. All columns consisted of slotted PVC pipes packed with a mixture (1:1 ratio by volume) of coarse grain Colorado silica sand (Carmeuse Natural Chemicals Industrial Sands; Pittsburgh, PA) and Rifle site sediment. The sediment from the site was collected by coring the subsurface prior to this study, stored at 4–6°C, and consisted of the unconsolidated floodplain materials typical of the site which were composed of feldspar and quartz sand, silts, and clays (Fox et al., 2012). A fraction of sediment used to fill the columns was separately preserved at −80°C on day 0 as a control. Each day, columns in the wells were briefly removed during geophysical inter-borehole acoustic monitoring (Saneiyan et al., 2019) and then replaced in the borehole after the monitoring. Due to this disturbance, we concurrently monitored the geochemistry of nearby undisturbed wells MLS-18 and MLS-21.

### Collection of Microbial Communities and DNA Extraction

To monitor both attached and free-living bacterial communities, sediment cores and groundwater were sampled, respectively. During the course of the field study, we aseptically collected 17 groundwater samples and six sediment samples to determine how aquifer microbial communities changed over time (Supplementary Table S2). For each sample, up to 2 L of groundwater (or until filter clogging occurred) was filtered through 0.22 μm pore-size Sterivex filters (Millipore, SigmaAldrich Inc., St. Louis, MO, United States). Filters were immediately frozen (liquid nitrogen) and stored on site until the end of the study when they were shipped frozen, overnight to Oregon State University and then stored at −80°C. DNA was extracted from each filter using the PowerWater DNA Isolation Kit (MoBio Laboratories Inc., Carlsbad, CA, United States). For sampling the sediments, the devices were removed from the wells, the sediment was aseptically extruded and then frozen in liquid nitrogen in the field. DNA was extracted from 0.25 g of sediment using the PowerSoil DNA Isolation Kit (MoBio Laboratories Inc.). Extracted DNA from filters and sediments was quantified using a Qubit^®^ 3.0 Fluorometer according to manufacturer’s instructions (ThermoFisher, Waltham, MA, United States).

### Community Composition

To determine microbial community composition in all samples, we sequenced the V4 hypervariable region of the 16S rRNA gene using the dual-barcoded primer pair 515F and 806R with added Illumina adaptor and barcodes sequences (Caporaso et al., 2011; Kozich et al., 2013). The final 20 μL PCR reaction mix consisted of 1X PCR Master Mix (Promega, Madison, WI, United States), 1 μL of template DNA, and 250 nM primers (final concentration). Cycling parameters were 94°C for 3 min; 35 cycles of 94°C for 45 s, 50°C for 60 s, 72°C for 90 s; 72°C for 10 min. Three technical replicates were sequenced per sample.

For groundwater, we cleaned the amplicons and normalized using SequalPrep Normalization Plates (ThermoFisher). The amplicons were sequenced using the Illumina MiSeq platform and v2 chemistry (2x250bp, paired end reads) at the Oregon State University Center for Genome Research and Biocomputing (OSU-CGRB). Sediment samples were cleaned using the QIAquick PCR Purification Kit (Qiagen, Germantown, MD, United States), and pooled at equimolar concentrations. Amplicons were sequenced as for the groundwater samples. Colorado River sediment adjacent to the Rifle site was used as an environmental control. Uninoculated sediment and no template controls from the MoBio Power Soil kit were used as negative controls.

We averaged all technical replicates and rarefied sequences to 3500 reads per sample (the rarefaction curves are shown in Supplementary Figure S3). We paired, quality controlled, and analyzed the sequences as in Crump et al. (2018) using QIIME and mothur. Sequencing reads were deposited in the National Center for Biotechnology Information (NCBI) Sequence Read Archive under accession #SRP150861.

### Ureolytic Functional Potential by *ureC* Gene Quantification

For all samples, we quantified the copy number of the *ureC* gene, a conserved subunit of the gene that codes for the urease enzyme. We used the QX200^TM^ AutoDG^TM^ Droplet Digital^TM^ PCR system (Bio-Rad, Temse, Belgium) at the OSU-CGRB. Genomic DNA from the ureolytic model organism *Sporosarcina pasteurii* was used as a positive control. Genomic DNA from *E. coli* K12 was used as a negative control. All samples were analyzed in triplicate on a PCR-clean 96-well plate (Eppendorf, Leuven, Belgium). Extracted DNA concentrations were determined by Qbit fluorometer and loaded to 1 μL/ddPCR rxn; except for samples with no detectable DNA, which were loaded with 2.5 μL of DNA of template/22 μL PCR rxn. The final 22 μL PCR reaction mixture contained 1X QX200^TM^ *ddPCR^TM^ EvaGreen^®^* Supermix, ∼1 ng of template DNA, and 0.5 μM primers (final concentration). We optimized cycling parameters for droplet digital PCR (ddPCR) with *ureC* gene-specific PCR primer pairs L2F/L2R (Gresham et al., 2007) with 94°C for 5 min; 40 cycles of 94°C for 1 min, 57°C for 1.5 min, 72°C for 3 min; 4°C for 5 min; 90°C for 5 min; 72°C for 15 min. These primers were recommended by Gresham et al. (2007) as being the most useful for detecting a range of aquifer microorganisms that possess the urease genes. After droplet generation and thermal cycling, we read the samples on a QX200 Droplet Reader and analyzed with Quantasoft (Bio-Rad) software.

### Statistical Analysis

We constructed a hierarchically clustered abundance plot using log transformed community composition data with the hclust function of the pheatmap package in R v.3.5. The dendrogram was generated using the hclust clustering algorithm with Euclidean distances. We also constructed a principal component analysis (PCA), with eigenvectors overlaid to explain where the microbial families laid in vector space using R v.3.5 and edited using Adobe Illustrator v. 22.1. OTU tables were used to calculate distance matrices, and phylogenetic diversity was calculated with Faith’s phylogenetic diversity metric (Faith, 1992).

To analyze the relationship between microbial taxa occurrence and abundance with a particular sample group, we used linear discriminant analysis (LDA) effect size (LEfSe) (Segata et al., 2011). LEfSe is an algorithm which determines “indicator taxa” most likely to explain differences between groups and considers both abundance and occurrence of particular taxa. It uses the Kruskal Wallis sum-rank test to detect differentially abundant features in a class of interest, using the Wilcoxon rank sum test and LDA to determine the feature effect size for that class. We defined the “feature” as the relative abundance of the 16S rRNA gene at family and OTU level, and the “class” as: (A) stage of the field study; with days 1–4 as “early,” days 5–10 as “mid,” and days 11–16 as “late”; (B) sample type; free living (water) and sediment; or (C) sample type in “late” stage; free living (water) and sediment.

To determine which taxa at the Rifle site had the capacity to be ureolytic, we compared this study’s microbial abundance with that from Anantharaman et al. (2016) and extracted all taxa with the urease gene present (Supplementary Figure S4).

### Linear Regression and Model Selection

To identify the best predictor variables of microbial species diversity (Shannon diversity) and ureolytic potential (based on the presence of the *ureC* gene), we used model selection using linear models (Grueber et al., 2011; Harrison et al., 2018). Two separate model selection processes were completed: one for species diversity as the response variable and one for ureolytic potential as the response variable. For each response variable, we tested for multicollinearity between all pairs of predictor variables. When high correlation coefficients (*r* > 0.70, Pearson) were found between two variables, we kept the variables that we hypothesized *a priori* would have the greatest effect on diversity or *ureC* presence. After selecting the most appropriate predictor variables, we performed model selection by ranking candidate models by Akaike Information Criterion (AICc), corrected for small sample sizes. A global model was created using all the predictor variables and interactions. The predictor variables were standardized to facilitate model convergence and interpretation of the parameter estimates using the arm package in R. The dredge function in the MuMIn package was used to find the top model(s) by comparing all the possible subsets of the global model. The global model and top models had normally distributed residuals. Model averaging was done to get parameter estimates from all the models within a delta AICc value of 2. Model weights of the top models are also presented. We used conditional averaging to get parameter estimates and relative variable importance. Analyses were conducted and lmerTest, lme4, Hmisc, nortest, arm, and MuMIn packages.

## Results

In this study, we used published methods to stimulate MICP in the aquifer (cf.,Colwell et al., 2005; Fujita et al., 2008; Smith et al., 2012) and measured changes in the microbial community structure, ureolytic potential, and chemistry occurring in the aquifer over time. We report evidence of microbial community dynamics, an increase in ureolytic potential, and successive oxidation and reduction of chemical species relevant to the progress of MICP in the system. These changes in the biogeochemistry of the system are linked to changes that were observed in the geophysical properties measured at the same time at the field site and detection of calcite precipitation occurring as a result of the MICP (Saneiyan et al., 2019).

### Microbial Community Structure

**FIGURE 2:**
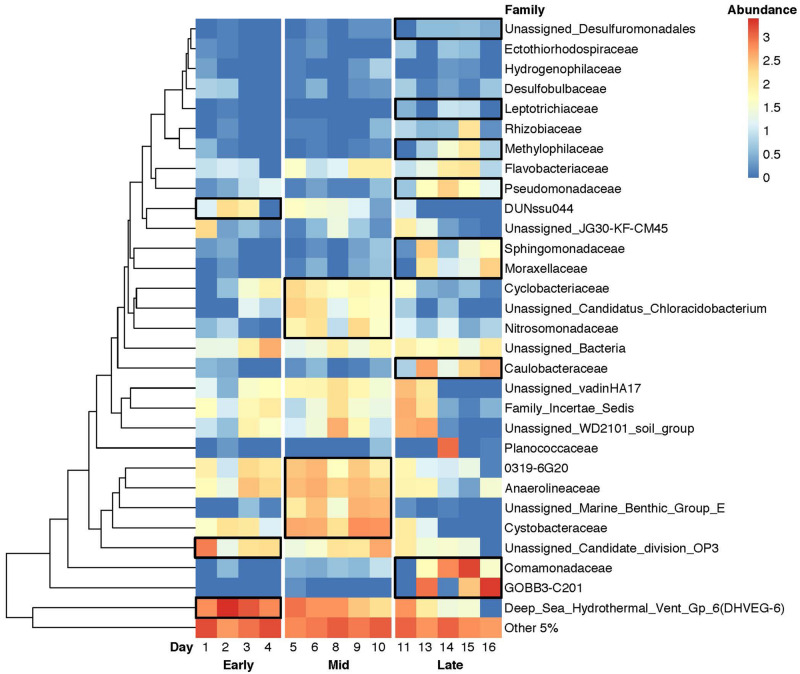
Heatmap of the relative changes in microbial community structure in groundwater from well LR-04 during the MICP field study. Dominant microbial families as determined by 16S rRNA gene sequencing are shown with colors representing the log of the relative abundance of rarefied copy number (3500 reads). Red and blue indicate higher and lower abundance taxa, respectively. Areas outlined by black lines indicate statistically significant indicator taxa for each “stage” (early, mid, late) of the study using the LEfSe algorithm.

Measurable changes in the aquifer microbial community structure were observed in groundwater and sediment samples during the study. Both groundwater and sediment communities shifted from diverse assemblages to *Proteobacteria-*dominated systems (Supplementary Figure S5). However, some *Proteobacteria* such as *DUNssu044* and myxobacteria *Cystobacteraceae* decreased with time in well LR-04 groundwater (Figure 2). The *Chloroflexi* group, previously determined to be dominant in the Rifle aquifer system (Hug et al., 2013) candidate division *OP3*, and *Euryarcheota* all decreased to less than 1% relative abundance by day 16. *Bacteroidetes* were present in every sample and increased in both groundwater and in columns over time. *Firmicutes*, naturally present in the aquifer in small proportion to the total community composition (Castelle et al., 2013; Supplementary Figure S5), increased to 29% of the well LR-04 microbial community by day 14, presumably due to the conditions associated with MICP. These changes co-occur with MICP-related geophysical and mineralogical observations at the same time and location in the aquifer. Saneiyan et al. (2019) report an increase in phase angle anomaly (a component of induced polarization analysis and useful for delineating MICP) across the aquifer at the location of LR-04 on day 14. Using X-ray diffraction, they also confirmed the presence of newly formed calcite in sediment cores extracted from LR-04 at the end of the study, and contrast this with the same sediments that were not incubated in the aquifer and do not have calcite.

Using LEfSe, we compared the groundwater microbial taxa (Supplementary Figure S6) and identified explanatory “indicator taxa” for each stage. Of those taxa, several microbes are known to have characteristic metabolic traits of interest to MICP. In the “early” stage, those taxa included *DUNssu044*, a member of the nitrogen-fixing *Rhizobiales*, and anoxic-associated candidate division *OP3* (Glöckner et al., 2010). In the “mid” stage, we found lithoautotrophic ammonia oxidizers *Nitrosomonadaceae* (Prosser et al., 2014) to be indicator taxa. The presence of these organisms is consistent with the presumed availability of ammonia in the aquifer as a result of urea hydrolysis (refer to the Introduction and the intended MICP reaction). In the “late” stage, we determined *Caulobacteraceae* to be indicator taxa. When we performed LEfSe on OTUs rather than family, we also found ureolytic taxa *Brevundimonas* (Wei et al., 2015) and calcite precipitating *Acinetobacter* (Zamarreño et al., 2009) to be indicator taxa for the “late” stage.

We also used LEfSe to compare groundwater to the incubated sediments and found that indicator taxa in the sediments included the biofilm-forming family *Pseudomonadaceae*, sulfur-oxidizing chemolithotroph *Thiobacillus* from the family *Hydrogenophilaceae* (known to have denitrifiers), and *DUNssu044* from the nitrogen-fixing order *Rhizobiales*, among other soil associated taxa. Other indicator taxa we identified by LEfSe include archaeal lineages MBG_E, MBG_B, and DHVEG_6. Archaea are present in the unaltered Rifle aquifer (Anantharaman et al., 2016) however, the functional traits for the taxa that we detected are uncertain as many of these taxa are uncultured and phenotypic traits are not yet confirmed.

### Microbial Diversity

**FIGURE 3:**
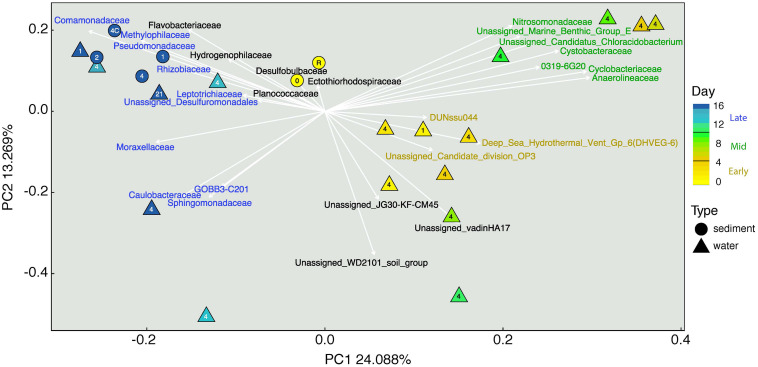
Principal component ordination of beta diversity for samples collected from sediments and groundwater during MICP field study. Eigenvectors (arrows) overlaid onto this plot show families that are most representative of samples of that vector space. Symbol shape corresponds to sample type (circles = sediments; triangles = groundwater) and color indicates the sample collection period. The number in the center of each symbol indicates the well from which the sample was obtained. Text color corresponds to significant taxa by LEfSe analysis where: yellow = “early” (0–4 days), green = “mid” (5–10 days), blue = “late” (11–16 days). Black colored taxa were not found to be significant indicator taxa by LEfSe.

Phylogenetic diversity was calculated by Faith’s index because this metric provides information about the diversity of communities and accounts for phylogeny, which can identify the taxa that characterize feature diversity (Faith, 1992). Phylogenetic diversity decreased from days 1 to 16 in both groundwater and in sediments (Supplementary Table S2). Notably, sediments incubated *ex situ* under diffusive flow were less diverse than those incubated *in situ* under advective flow. Beta diversity, a measure of diversity between samples, was measured using UNIFRAC distances and visualized using principal component analysis (PCA) to show the relative change of the communities during the MICP study period (Figure 3). PC axis 1 explained 24% of the variance, with samples collected during the field study appearing to be separated along this axis according to the different times that they were collected. PC axis 2 explained 13% of the observed variance in the samples and seemed to group samples more according to sample type (sediment vs. water) with sediment samples clustering closer to each other. Sediment samples grouped near samples taken at the same time period, with the more diverse initial sediment and water communities near each other, and the late phase water and sediment communities grouping tightly and even sharing indicator taxa such as *Comamonadaceae*, *Pseudomonadaceae*, *Methylophilaceae*, and *Caulobacteraceae*. Eigenvectors overlaid on the PCA plot explain the direction and magnitude of the variance of the microbial families most representative of the composition of the samples in PC space. We also found that several of the LEfSe indicator taxa (see Supplementary Figure S6) were also present as these microbial families overlaid in PCA vector space and grouped by stage of MICP treatment and sample type.

### Predictive Models for Diversity and *ureC*

Model selection was used to find the best set of predictor variables for microbial diversity (Shannon diversity, Table 1) and *ureC* copy number (ureolytic potential, Table 2). For diversity, the top models (achieving delta AICc values < 2) all contained the interaction between ammonia and *ureC*. This interaction term had the highest model weight (0.13) out of all possible models. The other models that were within 2 delta AICc values also included sulfate and pH. The model averaged parameter estimates show that *ureC* and the interaction between *ureC* and ammonia are significant predictors of microbial diversity. The variables with the highest relative importance are ammonia, *ureC*, and their interaction (Table 3). Ammonia by itself was not a significant predictor of diversity. Likewise, sulfate and pH were not significant in explaining microbial diversity.

**TABLE 1:** Models that most effectively predict microbial species diversity (Shannon Index) as a function of geochemistry and *ureC* gene copies using multivariate linear regression.

**Model**	**AICc**	**Delta AICc**	**Model weight**
ammonia * *ureC*	60.0	0.00	0.130
ammonia * *ureC* + sulfate	61.0	0.94	0.082
ammonia * *ureC* + sulfate + pH	61.1	1.10	0.075
ammonia * *ureC* + pH	61.9	1.84	0.052

*Only models with a delta AICc value < 2 are included in the model averaging and shown in the table. Model weight is the weight of all possible models. The multiple R^2^ for the top model presented (ammonia ^∗^*ureC*) is 0.52.*

**TABLE 2:** Models that most effectively predict *ureC* copy number (ureolytic potential) as a function of geochemistry using multivariate linear regression.

**Model**	**AICc**	**Delta AICc**	**Model weight**
ammonia	404.1	0.00	0.068
ammonia + bromide	404.3	0.17	0.062
Intercept	404.7	0.51	0.052
pH + ammonia + bromide	404.7	0.58	0.051
ammonia * bromide + pH	405.2	1.10	0.039
ammonia + bromide + DNA	405.6	1.43	0.033
ammonia * bromide + ammonia * pH	405.8	1.63	0.030
ammonia * pH + bromide	406.0	1.86	0.027
ammonia * pH + ammonia * sulfate	406.1	1.96	0.025
pH + ammonia	406.1	1.96	0.025

*Only models with a delta AICc value < 2 are included in the model averaging. Model weight is the weight of all possible models. The multiple R^2^ for the top model presented (ammonia) is 0.13.*

**TABLE 3:** Model averaged parameters for predicting microbial diversity (Shannon Index) using conditional averaging.

**Variable**	**Relative variable importance**	**B**	**se**	**z**	**P**
Intercept		3.82	0.15	23.26	<0.001
Ammonia	1.00	–0.65	0.43	1.43	0.15
*ureC*	1.00	–1.33	0.40	3.11	0.002
ammonia * *ureC*	1.00	1.68	0.46	3.41	<0.001
Sulfate	0.46	0.53	0.31	1.60	0.11
pH	0.38	0.60	0.39	1.42	0.15

*Parameters listed are only those that are in the top models (delta AICc < 2) shown in Table 1. B = estimate; se = standard error; z = z-value; p = p-value.*

The models that predict *ureC* most effectively all included ammonia, except the intercept only (null) model (Table 2). Other variables in the top models were bromide, pH, DNA, and sulfate. However, the intercept only model was just as good at predicting *ureC* than the models with variables. Therefore, the variables considered here do not seem to be important in predicting *ureC*. There is no model, out of all possible models, that is weighted above 0.10 (Table 2). The model averaged parameters are shown in Table 4. The variable with the highest relative importance is ammonia, followed by bromide, but these variables were not significant.

**TABLE 4:** Model averaged parameters for predicting *ureC* copy number (ureolytic potential) using conditional averaging.

**Variable**	**Relative variable importance**	**B**	**Se**	**z**	**P**
Intercept		1258.4	642.2	1.92	0.06
Ammonia	0.87	2605.8	1654.7	1.54	0.12
Bromide	0.59	–1388.3	853.6	1.54	0.12
pH	0.48	1715.5	1219.5	1.36	0.17
ammonia * bromide	0.17	–1552.3	890.6	1.62	0.10
DNA	0.08	–881.6	662.4	1.25	0.21
ammonia * pH	0.20	6093.7	4510.8	1.31	0.19
Sulfate	0.06	6393.5	2001.5	2.97	0.003
ammonia * sulfate	0.06	18078.7	5814.2	2.89	0.004

*Parameters listed are only those that are in the top models (delta AICc < 2) shown in Table 2. Variables as defined in Table 3.*

### Ureolytic Functional Potential (*ureC*)

Ureolytic potential was measured by determining the copy number of *ureC*, the conserved subunit of the functional gene urease. *E. coli* K12 was used as a negative control, and all measured values less than or equal to 36.4 copies/μL of the extracted sample were considered to be at the lower limit of detection for *ureC*. When *ureC* was adjusted per mL of filtered groundwater, *ureC* gene copies fluctuated from 0.07 to 3.07 copies/mL (not including samples below the detection limit). *ureC* copy numbers in water from LR-04 varied over the MICP study with a number of the samples yielding values that were below the limit of detection for the assay (Supplementary Table S2). Samples with these undetectable values also had undetectable levels of double-stranded DNA suggesting insufficient DNA in the samples. The highest values for *ureC* copy numbers were noted on day 14 (Supplementary Table S2) which was coincident with the period when *Firmicutes* dominated (Supplementary Figure S5). *ureC* copy number was highest in sediments incubated *ex situ*, under advective flow. These sediments also had lower phylogenetic diversity than sediments incubated *in situ* under diffusive flow. All sediments incubated in the monitoring wells had negligible levels of *ureC*. However, *ureC* was present in the sediments collected from the Colorado River bank. Phylogenetic diversity of communities from the river bank sediments was also high.

### Trait Analysis and Abundance Comparison of Rifle Communities

To determine which taxa at the Rifle site had the capacity to be ureolytic, we compared our study’s microbial abundance data with that from Anantharaman et al. (2016). From these, we extracted all taxa with the urease gene present (Supplementary Figure S4). We found a large proportion of these organisms that possessed urease genes also belonged to “late” phase indicator taxa (LEfSe) such as *Comamonadaceae* and *Pseudomonadaceae* (Figure 2). Interestingly, many taxa found in both Anantharaman et al. (2016) and this study were also positive for markers indicative of denitrification (NO_3_^–^ → N_2(g)_).

### Injectate Delivery and Geochemistry

The predominant groundwater flow, towards the Colorado River, was confirmed as bromide was detected in the downgradient wells, with the highest concentrations found in zones C, F, and D (see Figure 1). Bromide was also detected in well LR-01, indicating that some upgradient movement of the injectate likely occurred in the aquifer (Supplementary Figure S7C). While effective for tracking the general flow of groundwater and progress of the treatment, the bromide tracer results were not straightforward. Our study cannot account for fine-scale, localized mixing (e.g., discontinuous or lateral flow) or exchange with aquifer solids in the aquifer at the study site. Previous hydrological studies conducted at the Rifle site in the same geological formation as our study (Anderson et al., 2003; Yabusaki et al., 2007; Li et al., 2010; Fox et al., 2012) provide baseline information for our research.

Aquifer pH fluctuated in all zones but generally decreased (Supplementary Figure S7A). In well LR-04, pH increased from 7.6 to 8.1 until day 13, after which it decreased to 7.35. Conductivity, a measure of the concentration of ions in solution, generally increased, particularly during the last five days of the injection period (Supplementary Figure S7B). For the geochemistry measurements, the ions most closely related to the trend of an increase in conductivity were bromide [Br^–^], ferrous iron [Fe^2+^], and ammonia [NH_3_/NH_4_^+^] (Supplementary Figures S7C–E).

Chemical species measured in the aquifer samples during the study showed evidence of changes indicating biogeochemical oxidation and reduction. Nitrate was detected at or below a background of 230 μM (as noted for the site in Matthews, 1987) except for a peak on day 6 in zones A, B, and C (Supplementary Figure S7H). Sulfate fluctuated within background levels, except for events on days 3 and 6, when concentrations decreased in zones C, D, F, and in well LR-01. Then, for 4 days during the midpoint of the study, sulfate increased in zones C, D, and E, after decreasing in all zones. Nitrite, not normally present in the aquifer, appeared during the midpoint of the study and remained high until day 10.

Ferrous iron (Fe^2+^), is soluble in anoxic waters and generated by anaerobic iron-reducing bacteria (Lovley, 2013) and so can be used to indicate anoxic conditions. By day 4, Fe^2+^ could not be detected in the treated area of the aquifer, indicating that Fe^2+^ had likely been oxidized to low solubility Fe^3+^-bearing complexes (Supplementary Figure S7D). Fe^2+^ remained undetectable in all zones until day 11, three days after a large molasses injection. Then by day 14, dissolved Fe^2+^ concentrations increased in zones C and D, before eventually decreasing to pre-injection levels.

Ammonia, a byproduct of urea hydrolysis, was generally low during the early portions of the field study but then detected at high concentrations at the end of the study, and in some cases peaked at levels higher than the upper detection limit of 5544 μM for this assay (Supplementary Figure S7E). On day 16, ammonia was also detected in well LR-01, further indicating the likelihood that injections affected groundwater upgradient of the injection wells.

## Discussion

It is of paramount importance to understand microbiological and geochemical changes in order to successfully execute bioengineering projects in aquifers. Microbiological monitoring can help assess the progress of a field scale experiment, determine key attributes of a successful or failed program (National Research Council, 2000) and may help refine strategies that are needed for large scale projects. This project aimed to determine how microbial communities responded when molasses and urea were added to groundwater to enhance microbial production of calcite in an aquifer. Overall, we induced a substantial change to the aquifer system and noted the appearance of microbial taxa corresponding to geochemical changes indicative of the stimulation, as well as evidence that microbial communities possessed the potential for active ureolysis.

### MICP Changes Aquifer Microbes and Chemistry

**FIGURE 4:**
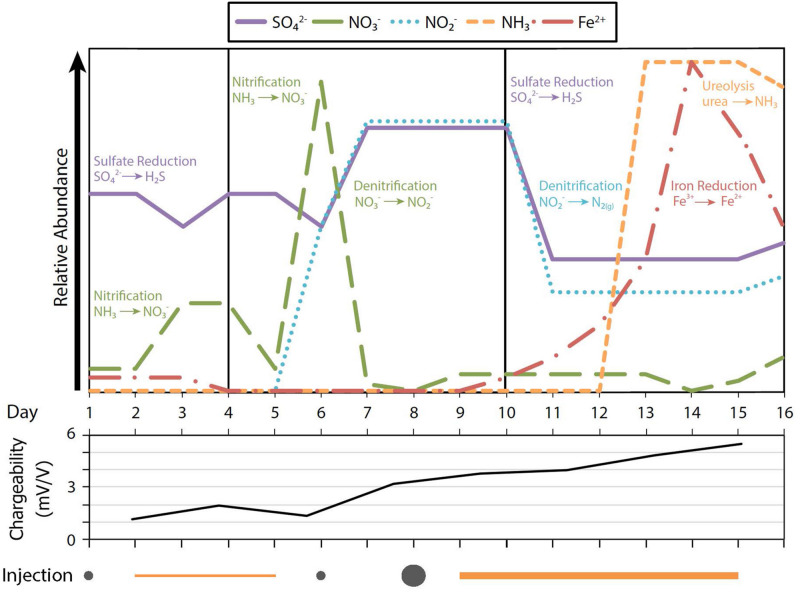
Synoptic changes in microbial processes and aquifer geochemistry (top panel) and corresponding changes in the geophysical property chargeability (as reported by Saneiyan et al., 2019 middle panel) over time during the MICP field study. Chargeability, a measure of the degree to which minerals can be electrically polarized, and known to be a good indicator of MICP, is shown for the area near LR-04 (details in Saneiyan et al., 2019). Nutrient injections are indicated (bottom panel) as gray circles for molasses and orange lines for urea, with increasing size or thickness, respectively, to indicate higher concentrations. The figure summarizes the responses observed throughout the aquifer at the field site and was formulated from data presented in Supplementary Figure S1.

MICP changed the groundwater microbial communities and geochemistry as determined by multiple measures (Figure 4). In some cases, those measures were consistent with what has been demonstrated in past MICP studies (e.g., an increase in ammonia concentration, and *ureC* gene abundance); however, some of our findings contrasted with our expectations. For example, we observed a decrease in groundwater pH whereas normally pH increases during MICP. In this section we describe the specific changes that we observed during our field experiment in the context of previous MICP investigations in the lab and field settings.

During the study, phylogenetic diversity of microbial communities decreased in both sediment and water samples indicating a significant change in the overall microbial structure. On day 14, groundwater from well LR-04 showed an abundance of *Firmicutes* (Supplementary Figure S5), a group previously shown to dominate laboratory experiments where microbial communities were examined over time in soils that were subjected to MICP (Gat et al., 2016; Dhami et al., 2017). Consistent with this shift in community structure, we noted that the highest levels of *ureC* gene in groundwater (per mL basis) also occurred at this time, a finding similar to that of Fujita et al. (2008) who detected a peak in *ureC* gene numbers at an intermediate time point of a MICP field study. The mid-study change in injection regime that we implemented may have had an effect on the microbial communities measured by the end of the experiment, for example, by providing more urea and stimulating an increase in urea hydrolysis potential. We cannot be certain that all possible urease-positive organisms were detected using this method but Gresham et al. (2007) comprehensively examined different urease primers for use in detecting such microbes and we used the primer set and conditions that they recommended. While a broad array of microbes from many environments possess the *ureC* gene and urease capability (Gresham et al., 2007) including many from the Rifle site (Anantharaman et al., 2016) a more detailed investigation at our MICP field site would be required to determine which specific microbes were associated with this function and which urea hydrolysis pathways dominate in the subsurface microbial communities.

Comparison of microbial numbers in sediments to those in groundwater is not always straightforward (Lehman, 2007). Nevertheless, our observations reflect a similar pattern of microbial community development for these different aquifer components. Coincident with an increased abundance of the taxa noted, we observed higher ammonia concentrations, presumably resulting from ureolysis, suggesting that the end of the study was an active period for urea hydrolysis and possibly MICP (Figure 4).

The Rifle aquifer has a diversity of microbes with a number of different respiratory and fermentative pathways; however, microbes likely to use oxygen and nitrate as electron acceptors were the dominant groups (Anantharaman et al., 2016). Changes in the aquifer geochemistry and microbial community properties signaled shifts in the availability of electron acceptors and donors (particularly among nitrogen and sulfur species) and in the types of microbes present. Ammonia concentration in the injectate tank was 89 μM (data not shown), close to background concentrations measured in the aquifer (< 38 μM; Matthews, 1987). The transient nitrate peaks during the “early” to “mid” stages may have resulted from microbial conversion of urea to ammonia and then from ammonia to nitrate. Introduction of dissolved oxygen in the amended groundwater may have encouraged this oxidation, followed by denitrification to nitrite, shown by the nitrite peak on days 7–9 (Figure 4). Subsequently, another urea addition on day 8 likely prompted a new phase of ureolysis as evidenced by higher levels of ammonia at the “late” stage (day 12 onward) of the field study. The levels of ammonia that we detected late in the study are similar to ca. 2 mM values reported by a previous MICP field study conducted at the Rifle site (Smith et al., 2012). Higher relative abundances of *Brevundimonas* (family *Caulobacteriaceae*) (Figure 2, Supplementary Figure S4) and *Planococcaceae* (phylum *Firmicutes*) were observed during this period. We have no explicit knowledge of the functional abilities of these groups. However, Gat et al. (2016); Dhami et al. (2017) also reported these microbes in lab studies promoting MICP. *Brevundimonas* were indicator taxa for the “late” stage (Supplementary Figure S6A) and *Planococcaceae* may have also been significant for the late stage as they became a larger fraction of the total community on day 14 (Supplementary Figure S5). Previous research at the Rifle site unrelated to MICP established the presence of the same taxa that we detected during our field study and trait analysis of their functional potential indicated that many possessed the capability for nitrogen-cycling (Anbu et al., 2016). The degree to which these microbes may metabolize nitrogen compounds, and could play a role in the corresponding peaks of nitrate, nitrite, and ammonia, was not examined in our research.

Addition of molasses at the beginning of the study to enhance microbial biomass coincided with a decrease in sulfate and detection of microbes that are typically understood to be sulfate-reducing bacteria (e.g., *Desulfobulbaceae*; Figure 4). A subsequent increase in sulfate concentrations, possibly due to sulfide oxidation, was noted, but by day 10 sulfate levels again decreased. Putative sulfate-reducing bacteria were again detected (*Desulfuromonadales* and *Desulfobulbaceae*; Supplementary Figure S6A).

The dynamics of iron chemistry that we observed during MICP may reflect the probable reduction of naturally occurring iron oxides in the system which may have occurred when a labile carbon source (i.e., molasses) was added, oxygen was consumed, and iron oxides became important as oxidants. Lee et al. (2020) observed this to occur in lab conditions that simulated MICP. Dissolved iron levels (Fe^+2^) were low until the second molasses addition on day 9 after which Fe^+2^ concentrations increased, peaking near day 14. After this point, Fe^+2^ concentrations began to decrease. The subsequent decrease in Fe^+2^ concentrations towards the end of the study may have been a result of iron oxidizing microbes.

In principle, according to expected reactions, ureolysis associated with MICP should cause an increase in alkalinity and pH (Colwell et al., 2005; DeJong et al., 2010). This has been demonstrated in lab studies with pure cultures as well as aquifer isolates known to be ureolytic (Fujita et al., 2000) and occurs in open systems where dissolved inorganic carbon (DIC) generated in the reaction can escape to the unsaturated zone as CO_2_. In contrast, we observed a decrease in pH in water samples collected during our study. A decrease in pH could result if DIC accumulates in an aqueous system (Stumm and Morgan, 1996). In the Rifle aquifer during the MICP study, increases in DIC may have occurred due to ureolysis itself, aerobic oxidation of organic compounds in molasses, or fermentation of molasses if dissolved oxygen was depleted. In each of these cases, if release of CO_2_ to the overlying vadose zone did not occur then the accumulation of DIC may have been responsible for the decrease in pH.

Other biologically mediated processes in the Rifle aquifer may have also contributed to the decrease in pH through the direct production of acids. *Nitrosomonadaceae*, which we detected and were abundant at the “mid” stage, can produce nitric acid (Crispim et al., 2004). All cultured representatives of this group are ammonium oxidizers and produce nitrate (Prosser et al., 2014). *Thiobacilli* were detected late in our study and some of these microbes can oxidize sulfides to form sulfuric acid which could lower pH. Acidic conditions can accelerate the release of sulfides from pyrite (Okabe et al., 2007; Vera et al., 2013) and pyrite is present in the Rifle aquifer (Qafoku et al., 2009). Fermentation of molasses to produce organic acids could also decrease pH, and microbial groups capable of fermentation were common in our samples and were reported by others who studied the Rifle site (Hug et al., 2013). As an example, *Anaerolineaceae*, a family of fermentative microbes in the phylum *Chloroflexi*, were abundant in Rifle sediments (Hug et al., 2013) and in our study at the “mid” stage. Though we cannot determine a specific cause of the decrease in pH, many processes occur simultaneously or sequentially in this complex system (Castelle et al., 2013; Yabusaki et al., 2017) and this could have been the case during our MICP experiment. Certainly, all of the aforementioned microbiological processes and the microbes capable of these metabolisms are present in the Rifle aquifer (Anantharaman et al., 2016; Yabusaki et al., 2017).

Our study at the Rifle site was conducted in parallel with non-invasive, real-time geophysical measurements of the treatment zone. Saneiyan et al. (2019) studied the complex electrical properties of the subsurface at Rifle using induced polarization (IP), and reported on how it changed as MICP developed in the aquifer as a result of the injections. The observed changes in IP signal correlate both spatially and temporally with the interfacial changes of subsurface materials in the treatment zone as caused by MICP and as depicted by a plot of chargeability (Figure 4), a measure of the magnitude by which minerals can be electrically polarized. This group also demonstrated the formation of calcite on mineral surfaces of our incubated artificial sediment columns, as measured by XRD, by the end of the field study (Saneiyan et al., 2019). The results from the geophysical study at our MICP field study are consistent with findings from a previous laboratory study in which IP detected and monitored progressive calcite precipitation and the associated increase in sediment shear velocity as evidence of increased mechanical strength of the sediment (Saneiyan et al., 2018). Saneiyan et al. (2019) also report lower hydraulic conductivities in the MICP treatment zone compared to an untreated zone as well as plugging of injection well 14 during the study suggestive of either calcite precipitation or biofilm formation in the aquifer. The MICP-related IP signal that they detected started at about day 6, and consistently increased in magnitude to the end of the study, corresponding to the changes that we report in the biogeochemistry of this system.

### MICP in a Heterogeneous, Complex System

As for many field studies in complex subsurface media, some of our observations are difficult to interpret, limited by the methods that we used, or indicative of an outcome that was not originally intended. One example was our inability to detect the conservative bromide tracer added to the injectate to record downgradient progress of injected fluids. Notable levels of bromide were only detected from day 10 until the end of the study in Zone C, which was downgradient from the injection wells (Figure 1B). The change in specific conductance (Supplementary Figure S7B) may have been due to introduced tracer ions, fermentation products from the molasses, or cation exchange processes on soil particle surfaces (Young and Crawford, 2004). The hydrology of the Rifle site in general is heterogeneous (Yabusaki et al., 2017) and a previous study using precisely the same field plot and wells as our MICP study reported complex flow paths in the aquifer with modeled flow velocities at different aquifer depths varying from each other by nearly an order of magnitude (Fox et al., 2012). We sampled selected wells at a single depth, rather than multiple depths as done by Fox et al. (2012) so we had limited sensitivity of bromide delivery in the system. We would not have detected bromide if it was present in a flow path that we did not sample.

Geochemical and microbiological responses upgradient of the injection wells and the target treatment zone indicated that our injection rates as well as biological responses must have affected the aquifer in the area near these wells. When molasses concentration was increased at a mid-point of the study, injection of fluids was somewhat impeded, possibly due to biological response to the additional nutrients. Also, during the study upgradient wells showed evidence of bromide tracer, an increase in specific conductance of the aquifer fluids, a decrease in pH, and ammonia above background levels, all indications that this area was also affected by the MICP treatment. Microbial composition of the upgradient groundwater and sediment was similar to downgradient well water and sediment, with a marked increase in *Proteobacteria*, as shown in Supplementary Figure S5. This, along with a decrease in phylogenetic diversity (Supplementary Table S2), suggests upgradient microbes also responded to the treatment.

In addition to the intended purpose of promoting MICP, addition of molasses could also have encouraged biofilm formation in the aquifer. Fujita et al. (2008) considered this possibility in a MICP field study. We have no direct evidence that biofilms formed in the aquifer; however, from days 3–4 and 10–16 we noted an increase in relative abundance of *Pseudomonas* spp., a taxon known to form biofilms (Bai et al., 2017). Certainly, biofilms alter aquifer flow paths (Cunningham et al., 2003) but an increase in biofilms can also create more nucleation sites for calcite precipitation (Decho, 2010; Dhami et al., 2017). More research related to the development of biofilms and calcite precipitation in MICP field projects is warranted.

### Considerations for Future MICP Research at Field Sites

MICP fits into a class of new geotechnical capabilities being considered to address a set of global problems (Culligan et al., 2019) and the details regarding the progress of MICP in an environmental setting will need to be resolved as for any newly implemented engineered processes. Some features of MICP may be classified as practical or engineering aspects of using the technology whereas others pertain more to enhancing a scientific or mechanistic understanding of fundamental processes evolving in such a system. In some cases, these classifications blur.

Future MICP field experiments should include a coring campaign to establish pre-existing conditions, and then target active MICP zones as identified by geophysical and well sampling. The study of cores collected before, during, and after MICP would allow direct observation of microbes on sediments (known to differ from those in water; cf., Lehman, 2007) detection of newly formed calcite in the subsurface, measurement of sediment strength, stiffness, and response to shear forces (Santamarina et al., 2019). Such analyses would complement data acquired by geophysical monitoring tools at the site (Saneiyan et al., 2019).

Establishing connections between salient changes in the microbial communities and changes in the geochemical and geophysical features of an aquifer or subsurface environment are at the forefront of studies that would move MICP towards application. Examples of these efforts still seem uncommonly applied in environmental settings (Scheibe et al., 2009; Reed et al., 2014) however, aspiring to a more comprehensive understanding of modified or evolving aquifers can help to move this science forward. From a microbial perspective, reaching beyond community characterization in samples to include direct measurement and quantitation of RNA transcripts or proteins for urease, carbonic anhydrase, or other enzymes implicated in successful MICP (e.g., nitrogen cycling) may result in detailed understanding of how the process is proceeding or identifying assays that report on success. Ultimately, practitioners will need a set of biological, chemical, and physical observations at a target MICP site that will help them assess progress and determine whether the manipulation is complete or requiring further amendment and assay. Building a repertoire of approaches will help to inform effective MICP strategies for commercial application (Phillips et al., 2013).

## Conclusion

As a geoengineered process, MICP has considerable potential for addressing a range of issues related to strengthening unconsolidated sediments, yet the technique must still be assessed for broad implementation so that key features regarding how the technology is applied in the field can be validated. Characterization of the changes in microbiological and geochemical properties of an aquifer submitted to MICP is a part of this validation. Our study determined that microbial taxa associated with ureolysis (the main metabolism that we sought to enhance), nitrification, denitrification, sulfate reduction, and iron reduction were present in the aquifer and responded rapidly as MICP progressed. Measured chemical signatures in the aquifer corresponded with metabolisms often associated with microbial communities in such systems. Consistent with successful MICP field implementation, our findings were corroborated by measurements of the electrical properties of aquifer solids and detection of precipitated calcite in sediment columns incubated *in situ* (Saneiyan et al., 2019). We also observed responses that could not easily be explained, some of which might be counter to the intent of MICP (e.g., a general decrease in aquifer pH); however, such occurrences are not unusual in complex natural settings and should lead to new sampling or analytical protocols needed to address questions that may arise. Further study of strategies to promote microbial precipitation of calcite to engineer aquifer and soil systems should be matched with appropriate methods to characterize microbial consortia and their activities so that the technology can progress.

## Data Availability Statement

The datasets generated for this study can be found in the National Center for Biotechnology Information (NCBI) Sequence Read Archive (SRA), accession number SRP150861.

## Author Contributions

JO, SS, JL, DN, SB, and FC designed the MICP study. JO, SS, JL, DN, and FC conducted the experiment in the field. AB performed statistical data analysis. The manuscript was written through contributions of all of the authors. All authors have given approval to the final version of the manuscript.

## Conflict of Interest

The authors declare that the research was conducted in the absence of any commercial or financial relationships that could be construed as a potential conflict of interest.

## Abbreviations

MICP: microbial induced calcite precipitation
OTU: Operational Taxonomic Unit.
