# Supplementary material for: Microbial and Geochemical Dynamics of an Aquifer Stimulated for Microbial Induced Calcite Precipitation (MICP)
Source: Front Microbiol. 2020 Jun 16;11:1327. doi: 10.3389/fmicb.2020.01327 (PMC7309221; doi:10.3389/fmicb.2020.01327)
Supplement: Supplementary file 1 [file Data_Sheet_1.PDF]

Supplementary Information

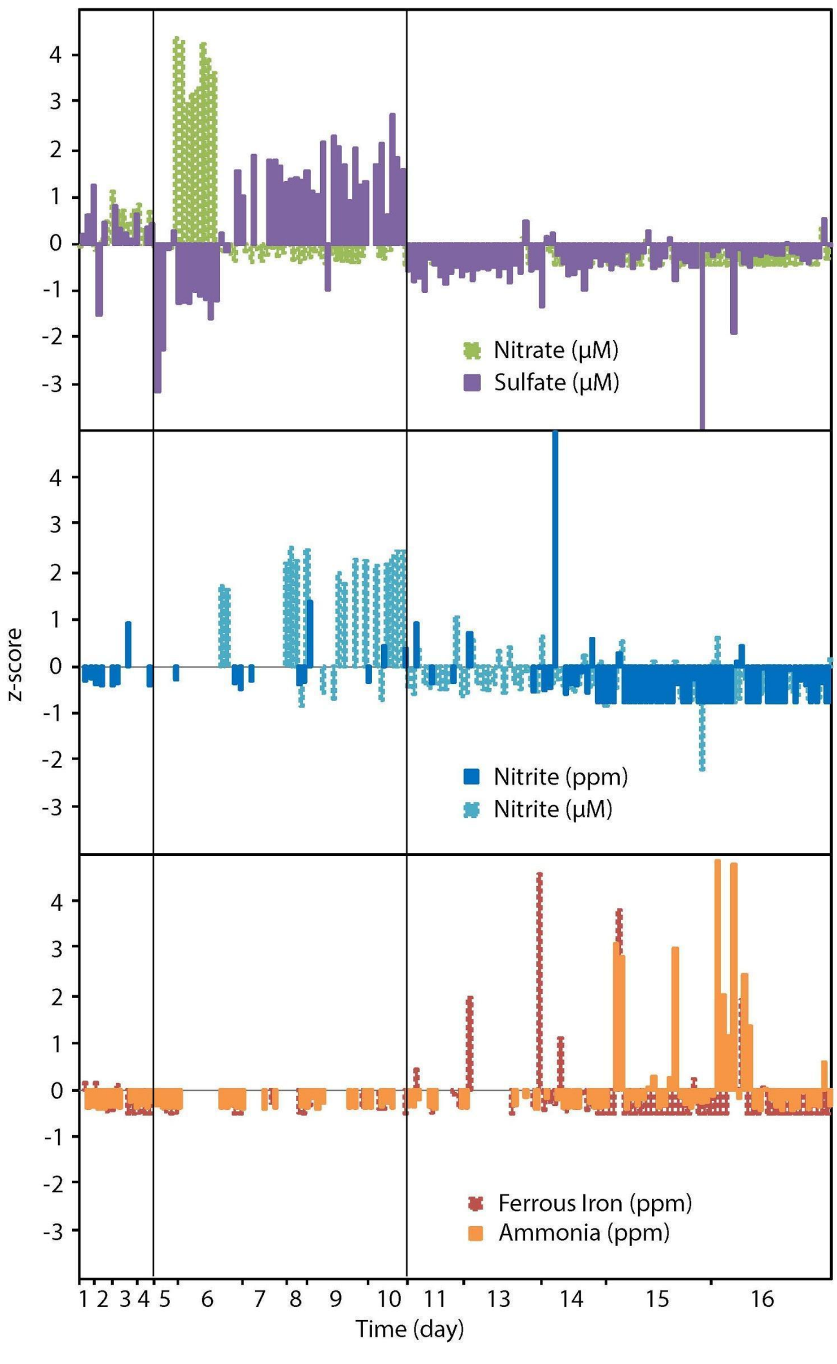

Figure S1. Transformed geochemistry (z-scores)
Z-scores of geochemical parameters over the 16-day study period. Values for all wells were plotted. Lines show partitioning into early" (day 0-4), "mid" (day 5-10), and "late" (day 11-16) time periods.

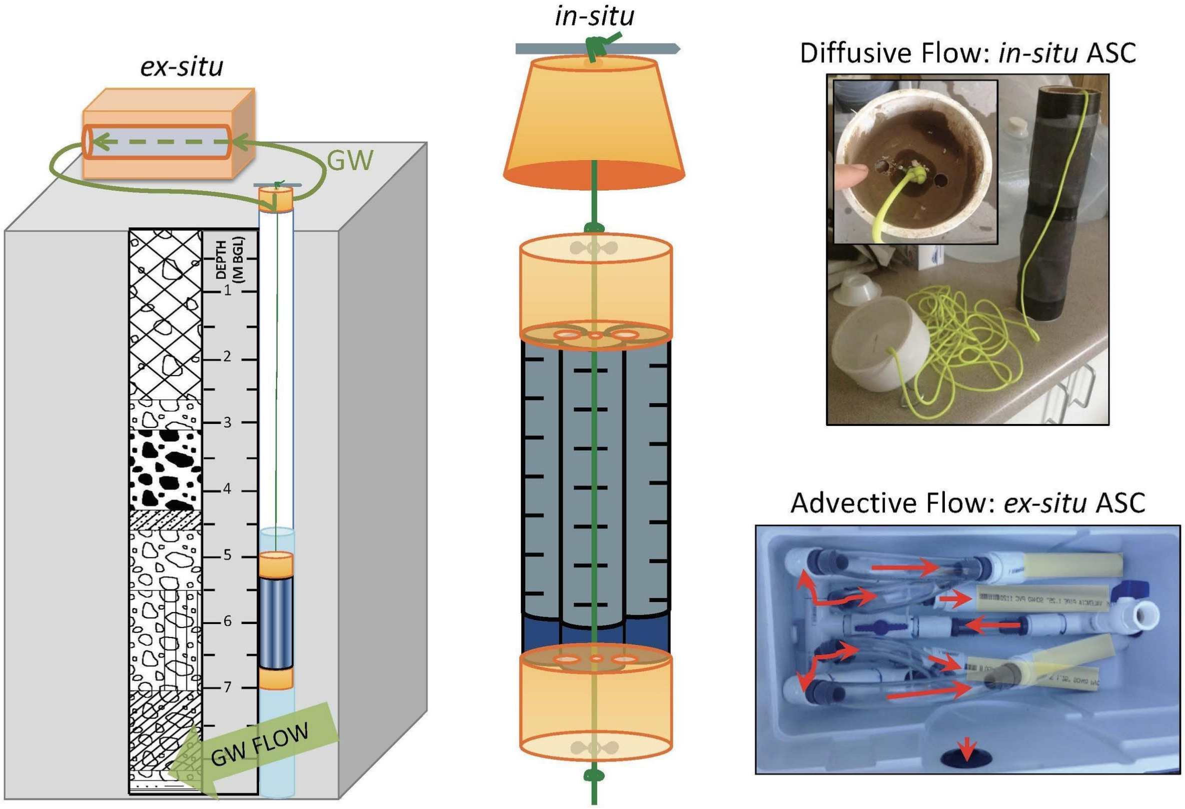

Figure S2. Artificial sediment core schematics.
In-situ artificial sediment cores were composed of three slotted PVC tubes (ID: 1.25in, L: ~10 in, slots: 1 in spacing), each holding ~200g of sediment. Cores were stoppered with synthetic resin-treated polymer pad (Scotch-Brite 3M, St. Paul, MN), and covered in small diameter nylon mesh to prevent sediment loss while allowing groundwater perfusion. PVC end caps (D: 3.25in) were drilled with two 0.5in holes to relieve vacuum pressure, and one 0.25in center hole to thread through 20ft, 110lb carrying load paracord. End caps were inverted and PVC tubes were zip-tied internally before being covered with large diameter vinyl mesh and secured with electrical tape. Ex-situ cooler sediment cores had no slots, and outflow was stoppered with synthetic scouring pads.

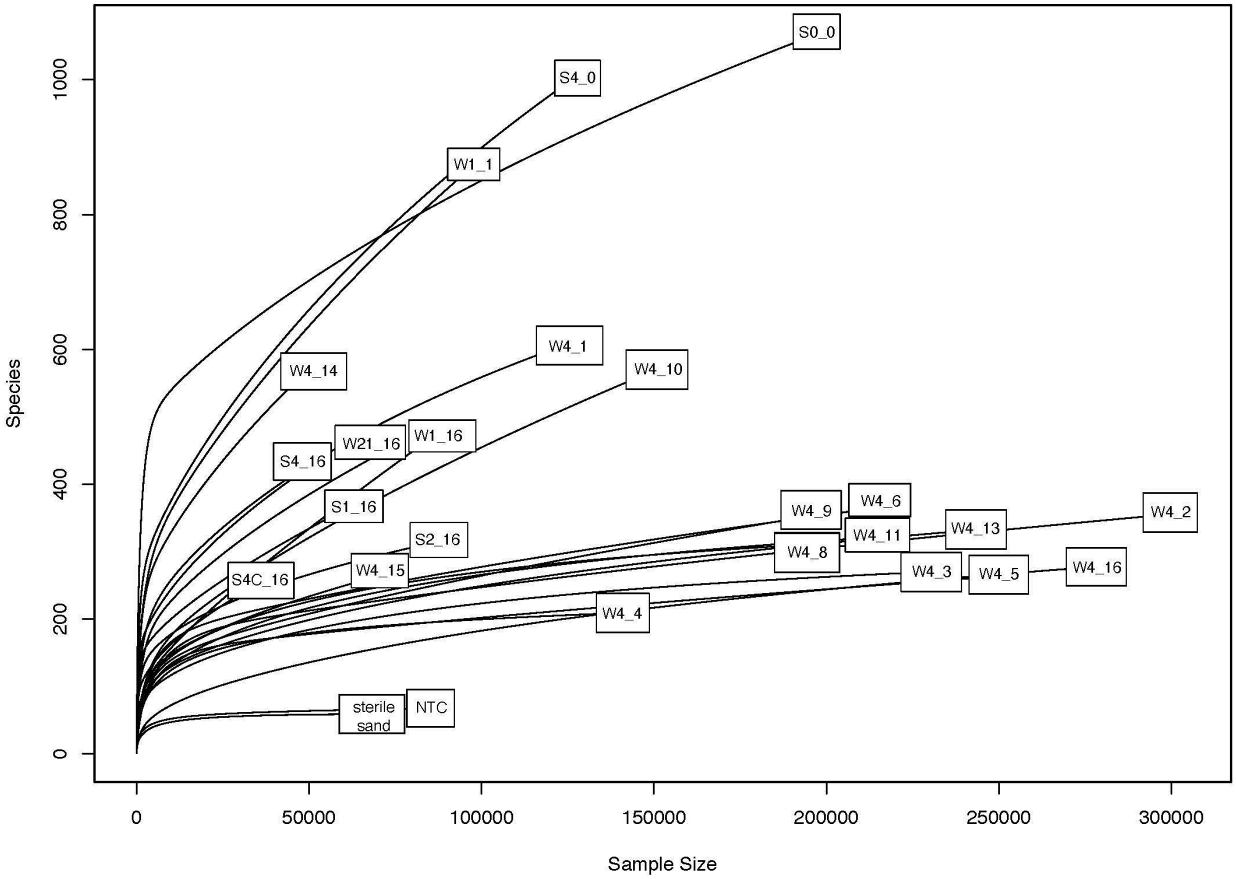

Figure S3. Rarefaction curves for groundwater and sediment samples, labeled as in Table S2. NTC = No template control.

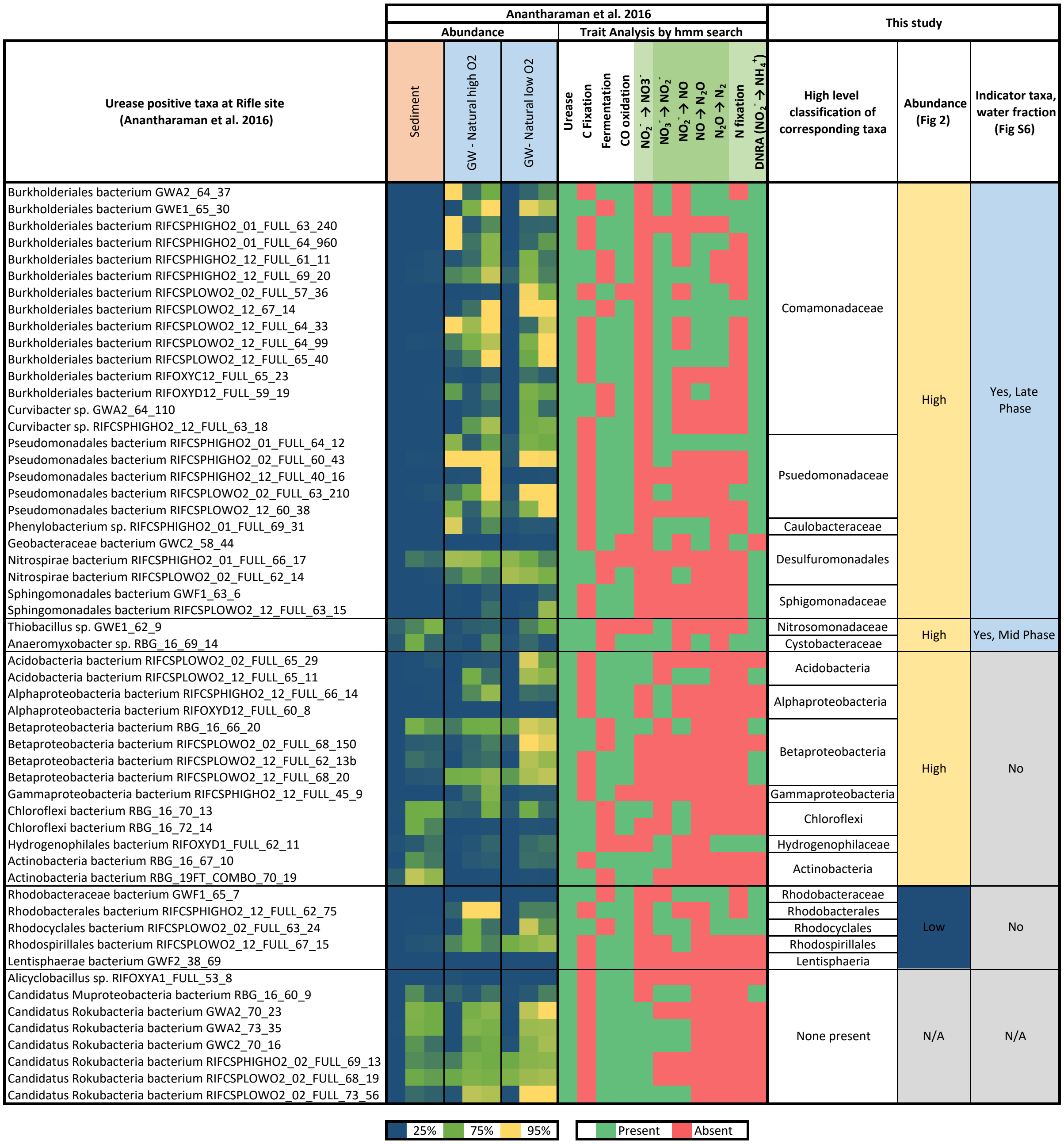

Figure S4 Trait analysis by hmm search for *urease*-positive organisms from Anantharaman et al. 2016 compared to this study. Abundance of the genomes found by Anantharaman et al. 2016 are measured as relative coverage in the microbial community at the Rifle site (sediment: RBG13, 16, 19; groundwater with high O2: RIFCSPHIGHo2_01, 02, 12; groundwater with low O2: RIFCSPLOWo2_01, 02, 12). Trait analysis genes were determined by hmm searches of marker genes and ggKbase lists for specific pathways associated with those traits by Anantharaman et al. 2016. We compare these data against taxa we found in our study that were present in high abundance, low abundance or absent, and if they were determined by LEfSe to be indicator taxa for certain phases. For more detailed abundance data from our study see Figure 2.

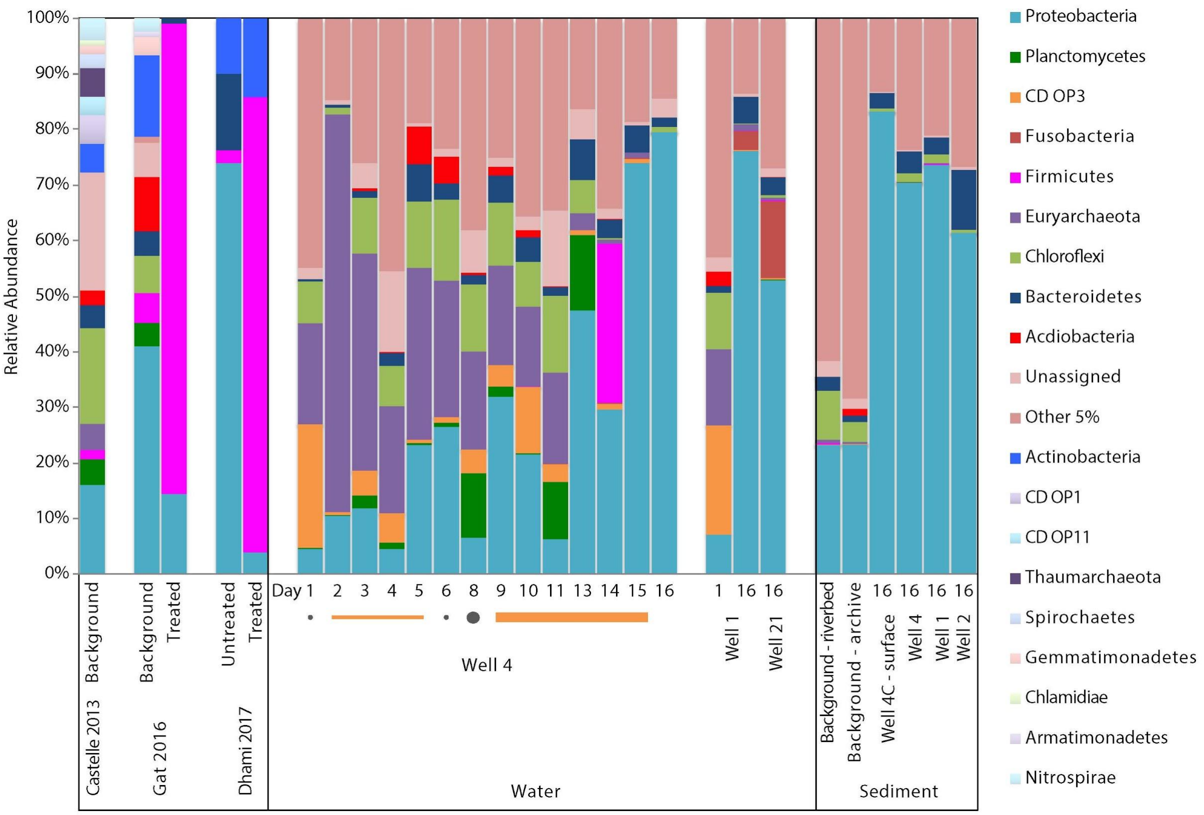

Figure S5. Microbial community structure (by phylum) of groundwater and artificial sediment. Phylum level microbial community structure of all samples collected from groundwater and sediment columns, using 16S rRNA gene sequencing. Presented as relative abundance of rarefied copy number (3500 reads). We also compare this study's community composition to laboratory scale MICP biostimulation studies that experimentally induced calcite precipitation using urea and molasses (Gat 2016) or urea and yeast extract (Dhami 2017) as a carbon source. We also include the community composition of untreated cored sediment of the Rifle site (Castelle 2013). Nutrient injections are indicated as grey circles for molasses and orange lines for urea, with increasing size or thickness, to indicate higher concentrations (See table S1 for exact concentrations).

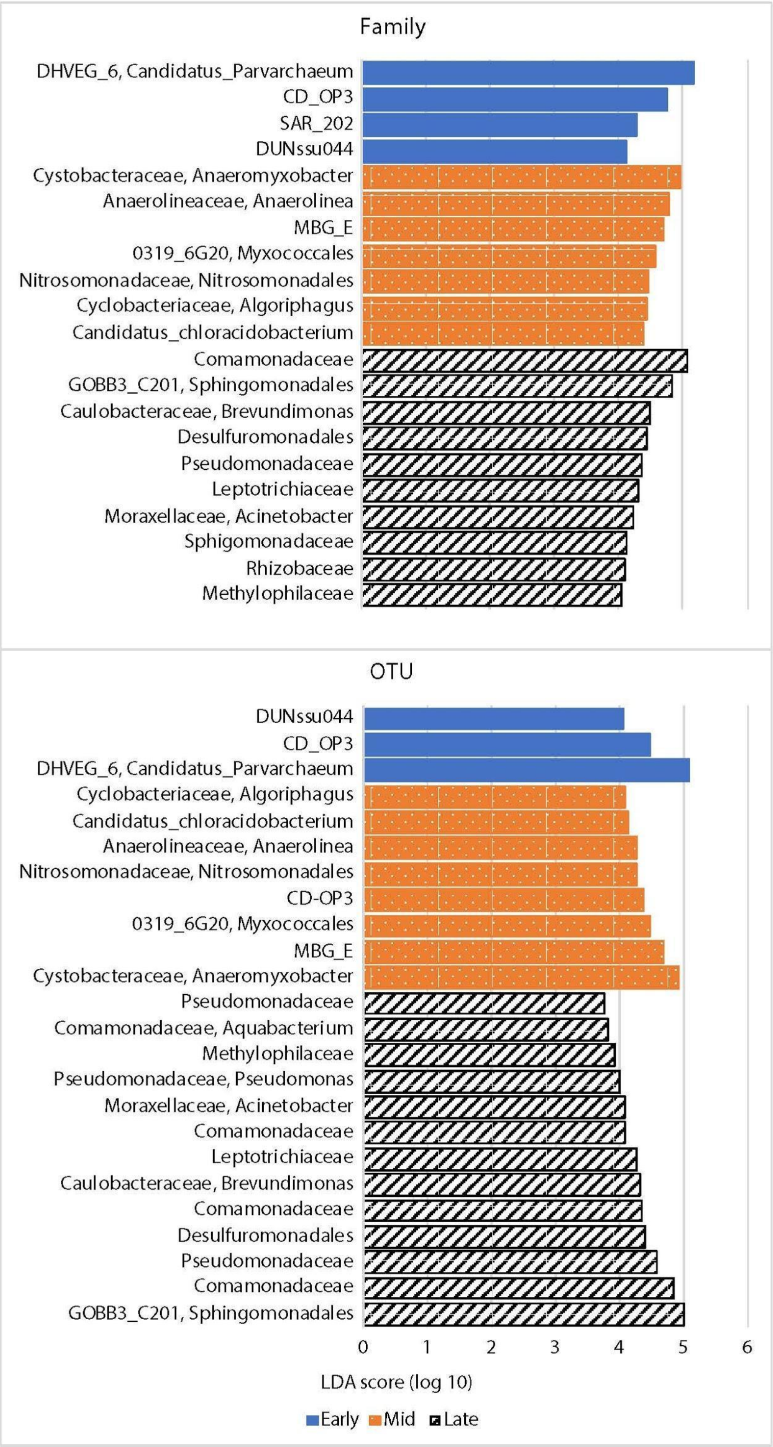
Family
DHVEG_6, Candidatus_Parvarchaeum
CD_OP3
SAR_202
DUNssu044
Cystobacteraceae, Anaeromyxobacter
Anaerolineaceae, Anaerolinea
MBG_E
0319_6G20, Myxococcales
Nitrosomonadaceae, Nitrosomonadales
Cyclobacteriaceae, Algoriphagus
Candidatus_chloracidobacterium
Comamonadaceae
GOBB3_C201, Sphingomonadales
Caulobacteraceae, Brevundimonas
Desulfuromonadales
Pseudomonadaceae
Leptotrichiaceae
Moraxellaceae, Acinetobacter
Sphigomonadaceae
Rhizobaceae
Methylophilaceae
OTU
DUNssu044
CD_OP3
DHVEG_6, Candidatus_Parvarchaeum
Cyclobacteriaceae, Algoriphagus
Candidatus_chloracidobacterium
Anaerolineaceae, Anaerolinea
Nitrosomonadaceae, Nitrosomonadales
CD-OP3
0319_6G20, Myxococcales
MBG_E
Cystobacteraceae, Anaeromyxobacter
Pseudomonadaceae
Comamonadaceae, Aquabacterium
Methylophilaceae
Pseudomonadaceae, Pseudomonas
Moraxellaceae, Acinetobacter
Comamonadaceae
Leptotrichiaceae
Caulobacteraceae, Brevundimonas
Comamonadaceae
Desulfuromonadales
Pseudomonadaceae
Comamonadaceae
GOBB3_C201, Sphingomonadales
0
1
2
3
4
5
6
LDA score (log 10)
Early
Mid
Late

A.

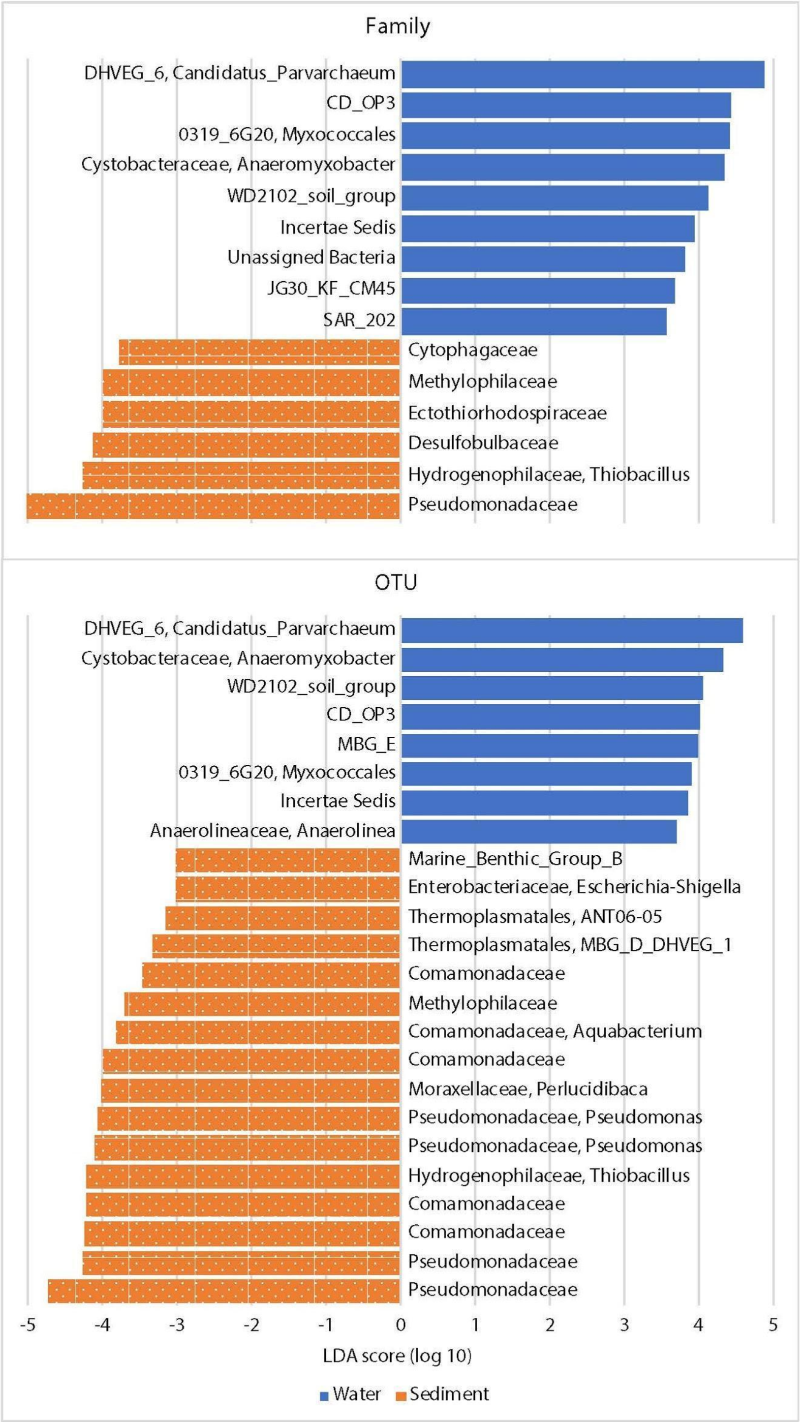
Family
DHVEG_6, Candidatus_Parvarchaeum
CD_OP3
0319_6G20, Myxococcales
Cystobacteraceae, Anaeromyxobacter
WD2102_soil_group
Incertae Sedis
Unassigned Bacteria
JG30_KF_CM45
SAR_202
Cytophagaceae
Methylophilaceae
Ectothiorhodospiraceae
Desulfobulbaceae
Hydrogenophilaceae, Thiobacillus
Pseudomonadaceae
OTU
DHVEG_6, Candidatus_Parvarchaeum
Cystobacteraceae, Anaeromyxobacter
WD2102_soil_group
CD_OP3
MBG_E
0319_6G20, Myxococcales
Incertae Sedis
Anaerolineaceae, Anaerolinea
Marine_Benthic_Group_B
Enterobacteriaceae, Escherichia-Shigella
Thermoplasmatales, ANT06-05
Thermoplasmatales, MBG_D_DHVEG_1
Comamonadaceae
Methylophilaceae
Comamonadaceae, Aquabacterium
Comamonadaceae
Moraxellaceae, Perlucidibaca
Pseudomonadaceae, Pseudomonas
Pseudomonadaceae, Pseudomonas
Hydrogenophilaceae, Thiobacillus
Comamonadaceae
Comamonadaceae
Pseudomonadaceae
Pseudomonadaceae
-5 -4 -3 -2 -1 0 1 2 3 4 5
LDA score (log 10)
Water Sediment

B.

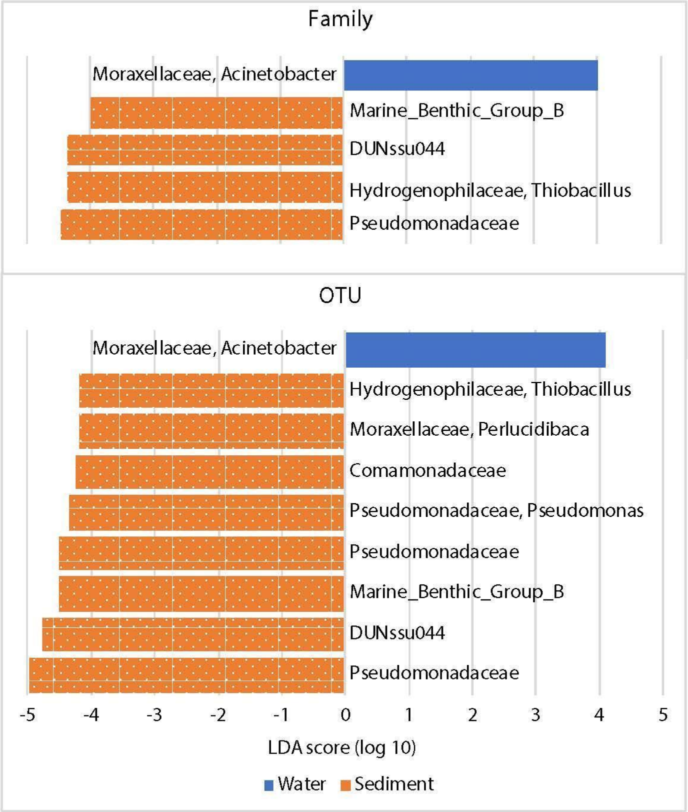

C.

Figure S6 A-C. Indicator taxa as determined by LEfSe analysis
LEfSe output showing indicator taxa when “class” is defined as (A) Water samples at all stages where “early” = day 1-4, “mid” = day 5-10, and “late” = day 11-16. (B) water and sediment samples overall, and (C) “late” stage water and sediment samples.

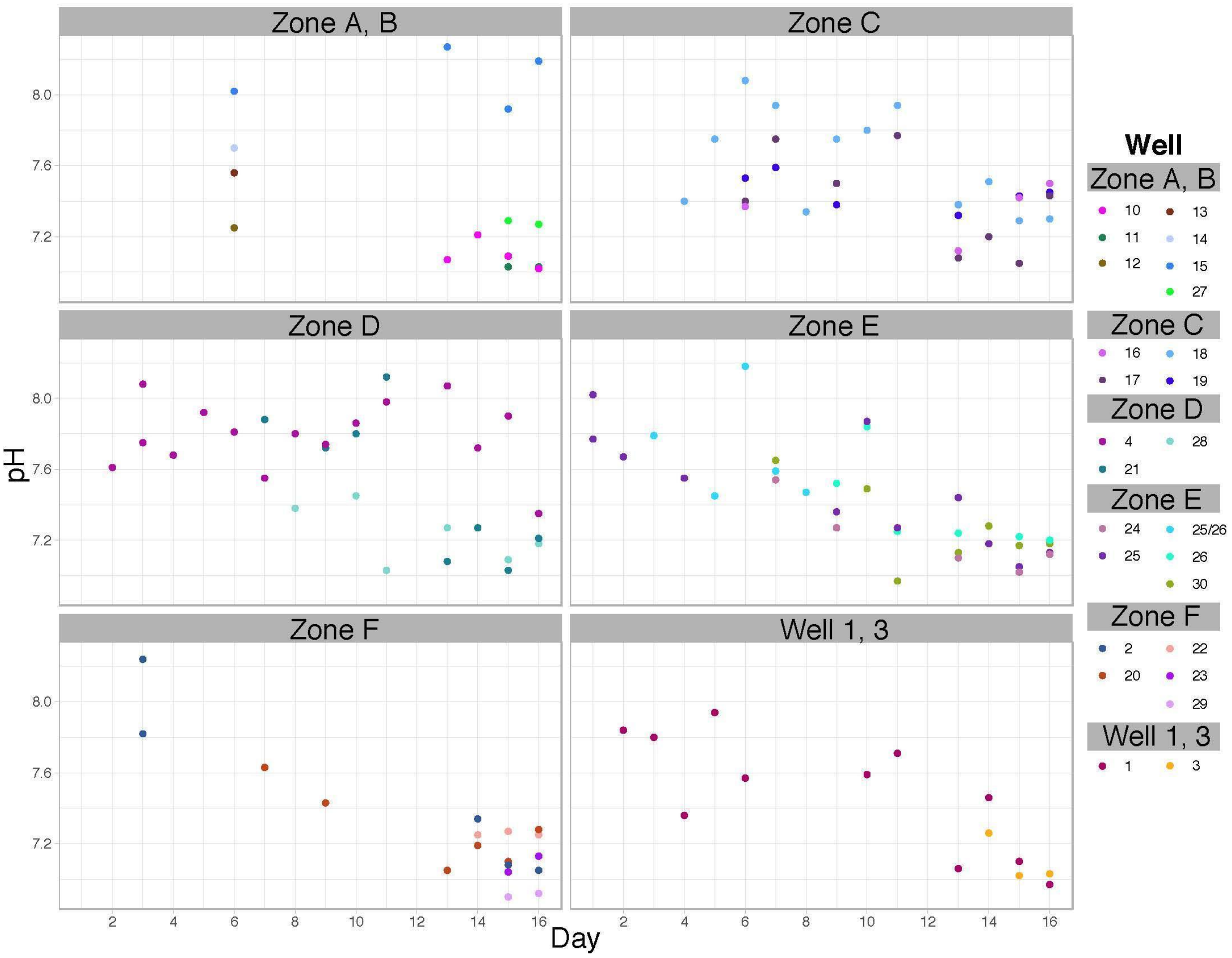

Zone A, B
Zone C
Zone D
Zone E
Zone F
Well 1, 3
pH
Day
8.0
7.6
7.2
2
4
6
8
10
12
14
16
Well
Zone A, B
10
13
11
14
12
15
27
Zone C
16
18
17
19
Zone D
4
28
21
Zone E
24
25/26
25
26
30
Zone F
2
22
20
23
29
Well 1, 3
1
3

A.

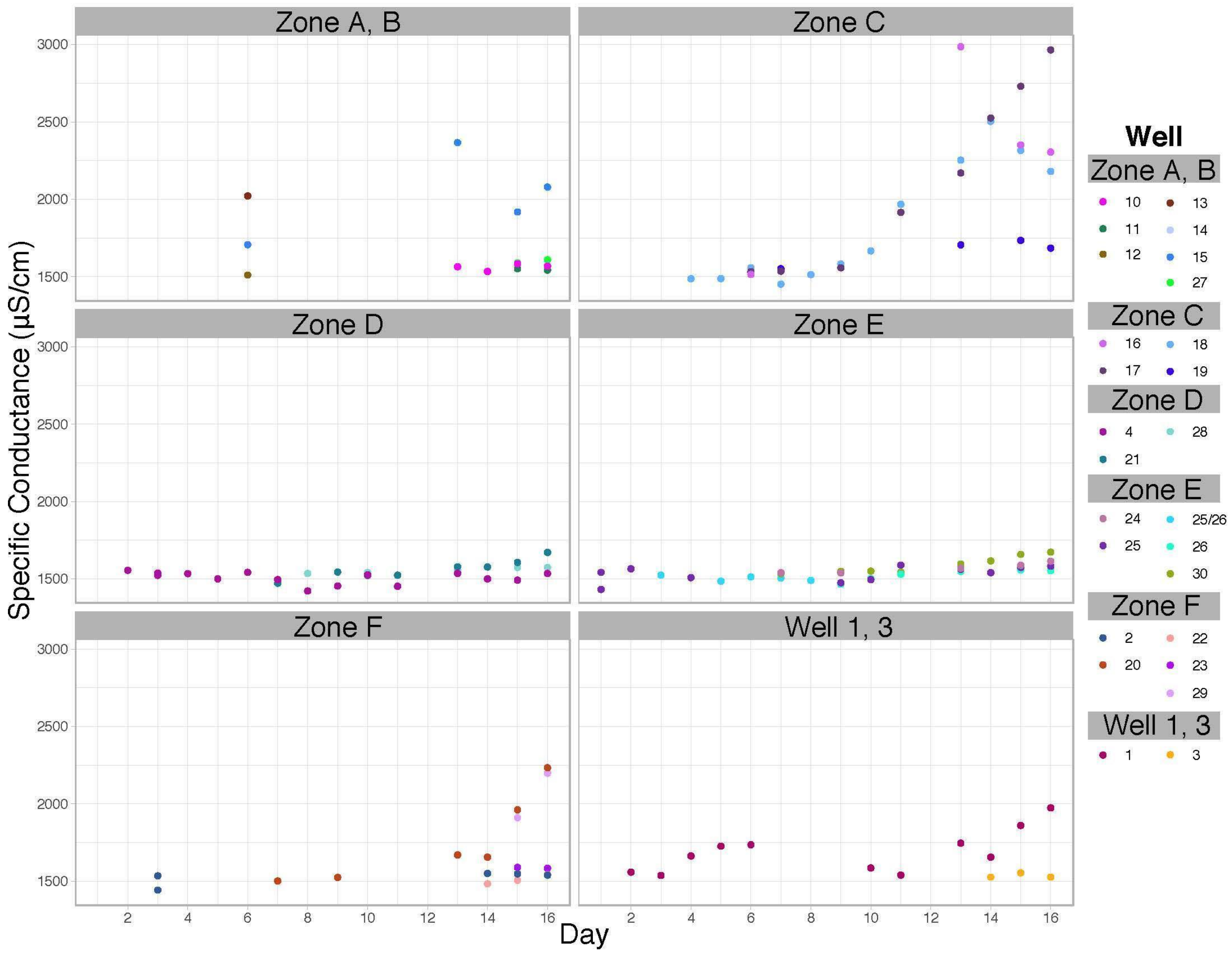

Zone A, B
Zone C
Zone D
Zone E
Zone F
Well 1, 3
Specific Conductance (µS/cm)
3000
2500
2000
1500
Day
2
4
6
8
10
12
14
16
Well
Zone A, B
10
13
11
14
12
15
27
Zone C
16
18
17
19
Zone D
4
28
21
Zone E
24
25/26
25
26
30
Zone F
2
22
20
23
29
Well 1, 3
1
3

B.

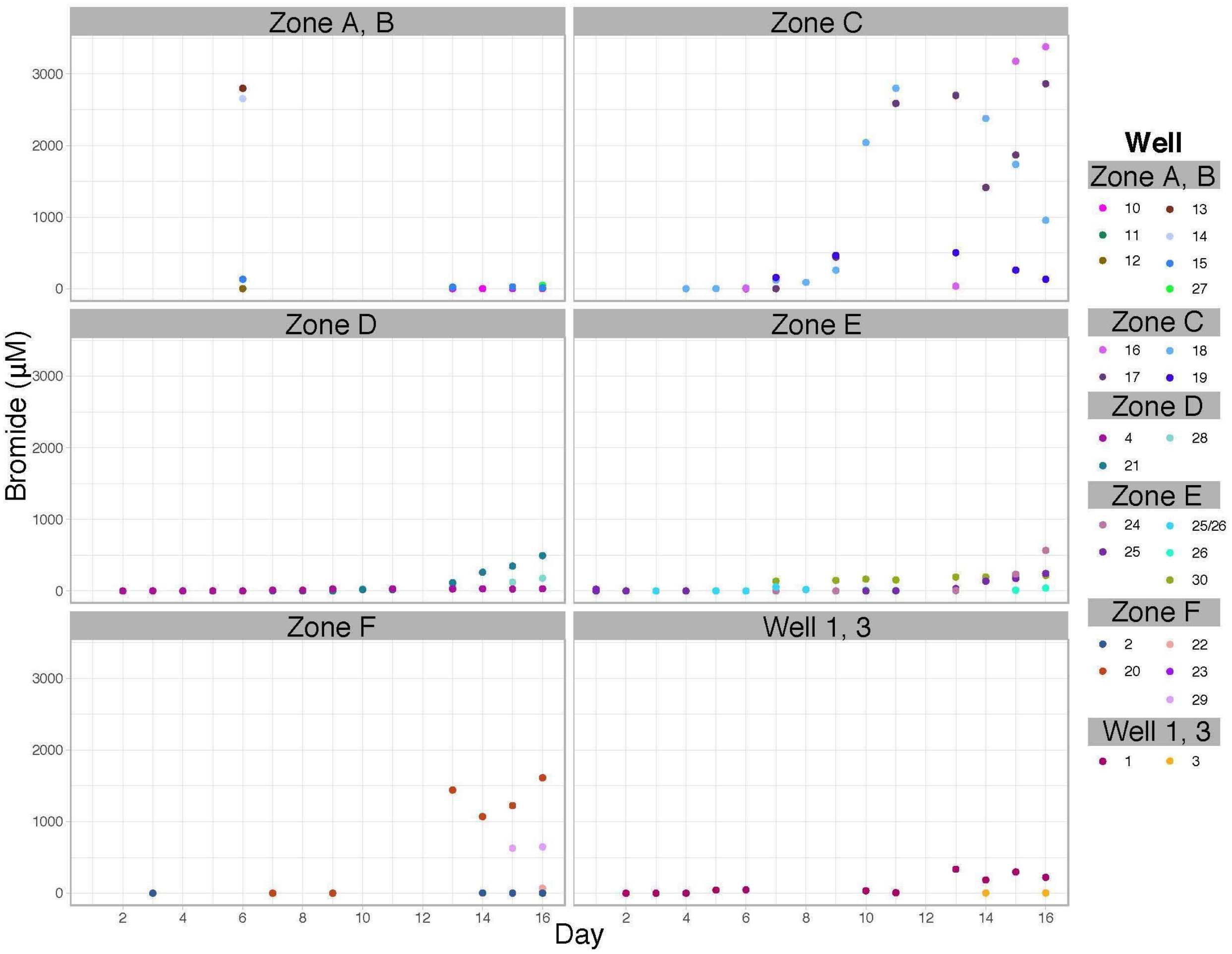
Zone A, B
Zone C
Zone D
Zone E
Zone F
Well 1, 3
Bromide (μM)
Day
0
1000
2000
3000
2
4
6
8
10
12
14
16
Well
Zone A, B
10
11
12
13
14
15
27
Zone C
16
17
18
19
Zone D
4
21
28
Zone E
24
25
25/26
26
30
Zone F
2
20
22
23
29
Well 1, 3
1
3

C.

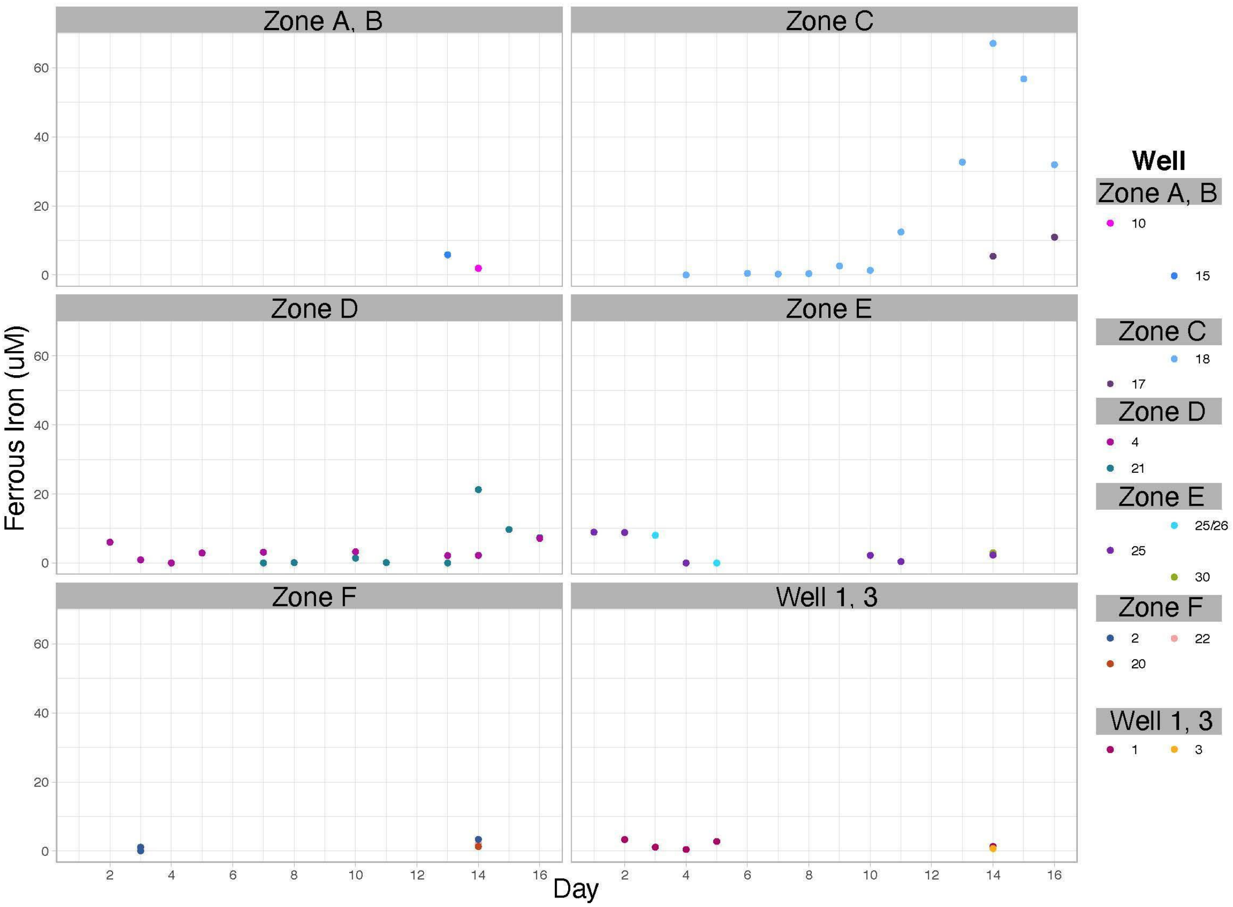
Zone A, B
Zone C
Zone D
Zone E
Zone F
Well 1, 3
Ferrous Iron (uM)
Day
0
20
40
60
2
4
6
8
10
12
14
16
Well
Zone A, B
10
15
Zone C
18
17
Zone D
4
21
Zone E
25/26
25
30
Zone F
2
22
20
Well 1, 3
1
3

D.

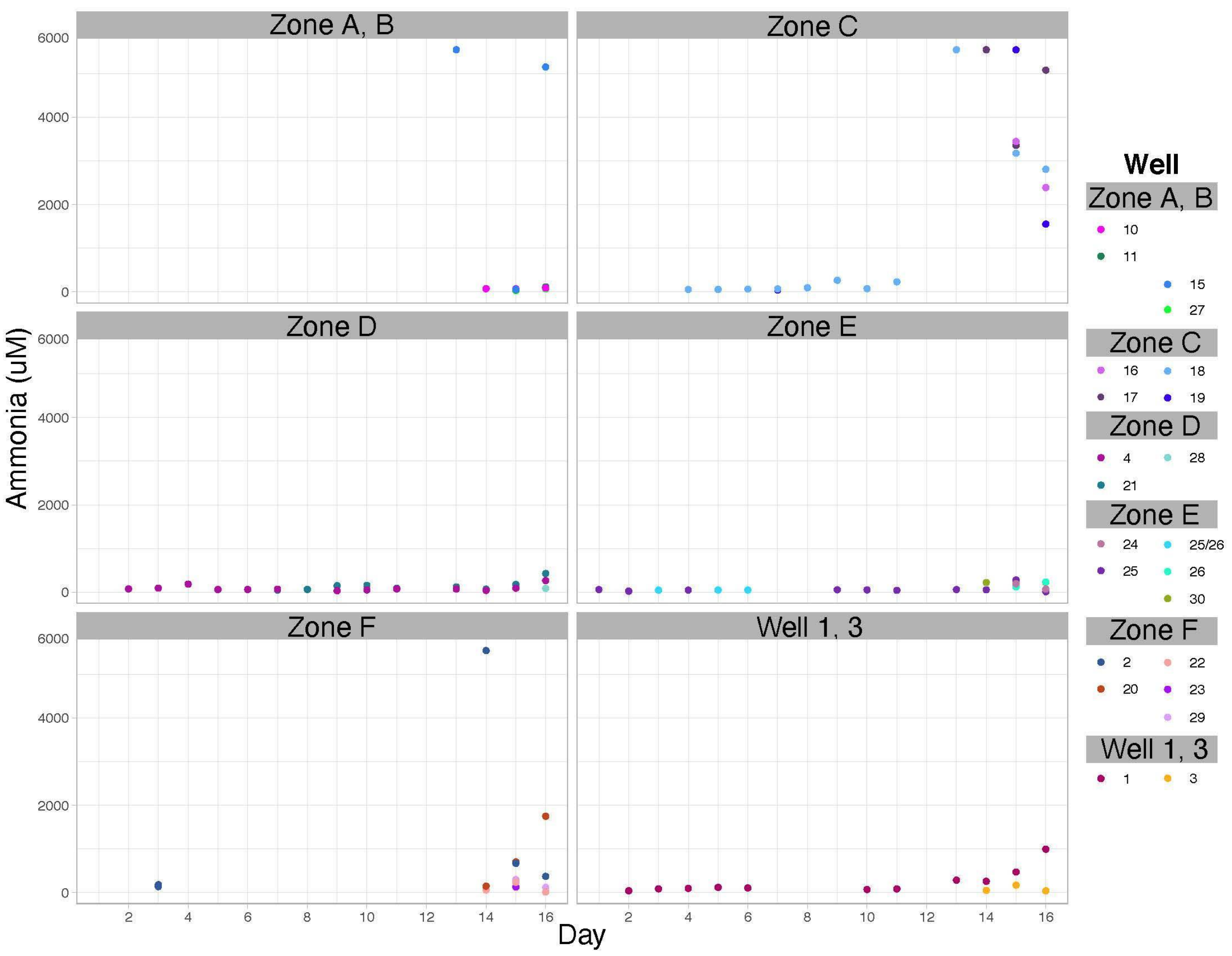
Zone A, B
Zone C
Zone D
Zone E
Zone F
Well 1, 3
Ammonia (uM)
Day
Well
Zone A, B
10
11
15
27
Zone C
16
18
17
19
Zone D
4
28
21
Zone E
24
25/26
25
26
30
Zone F
2
22
20
23
29
Well 1, 3
1
3

E.

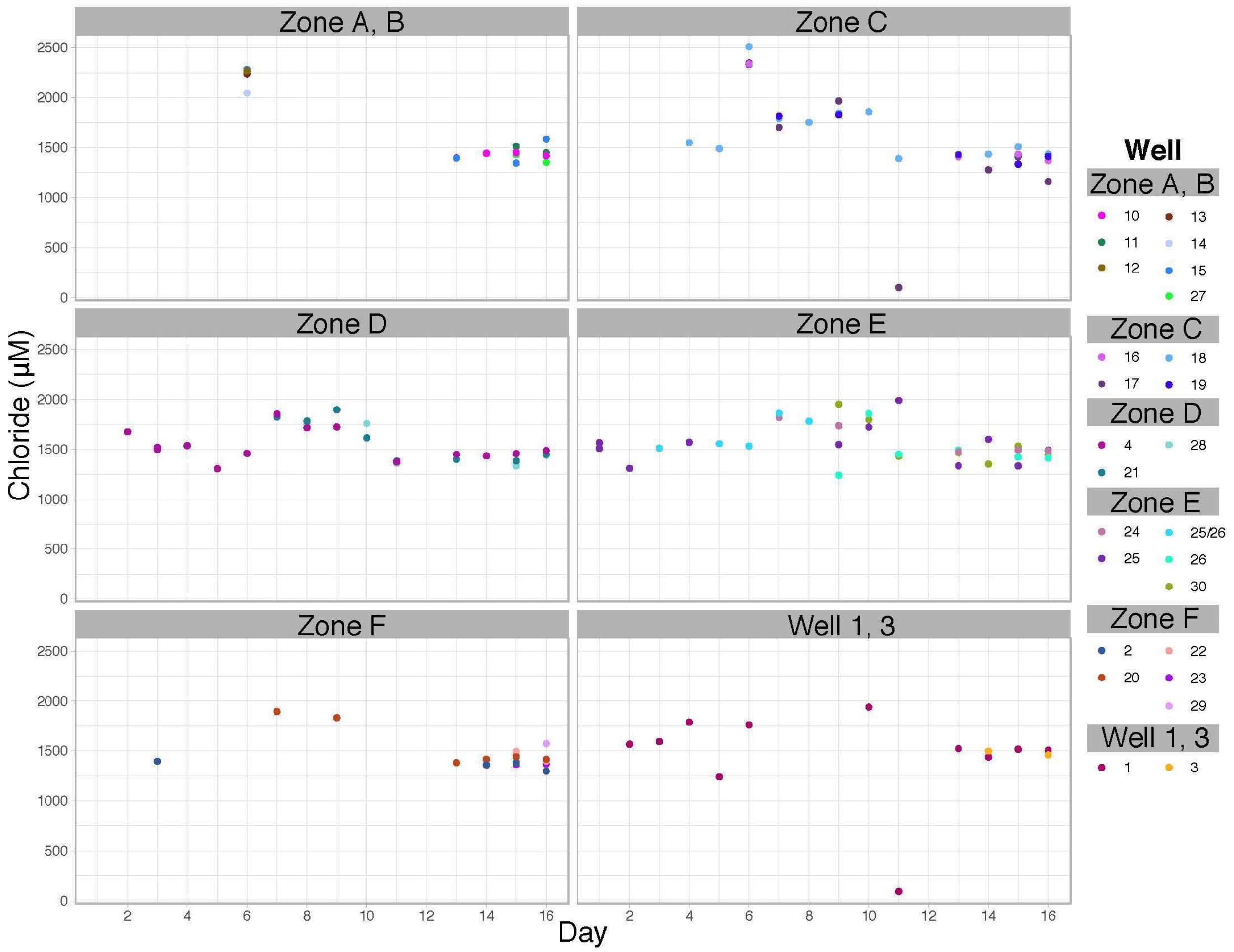

Zone A, B
Zone C
Zone D
Zone E
Zone F
Well 1, 3
Chloride (µM)
Day
Well
Zone A, B
10
11
12
13
14
15
27
Zone C
16
17
18
19
Zone D
4
21
28
Zone E
24
25
25/26
26
30
Zone F
2
20
22
23
29
Well 1, 3
1
3

F.

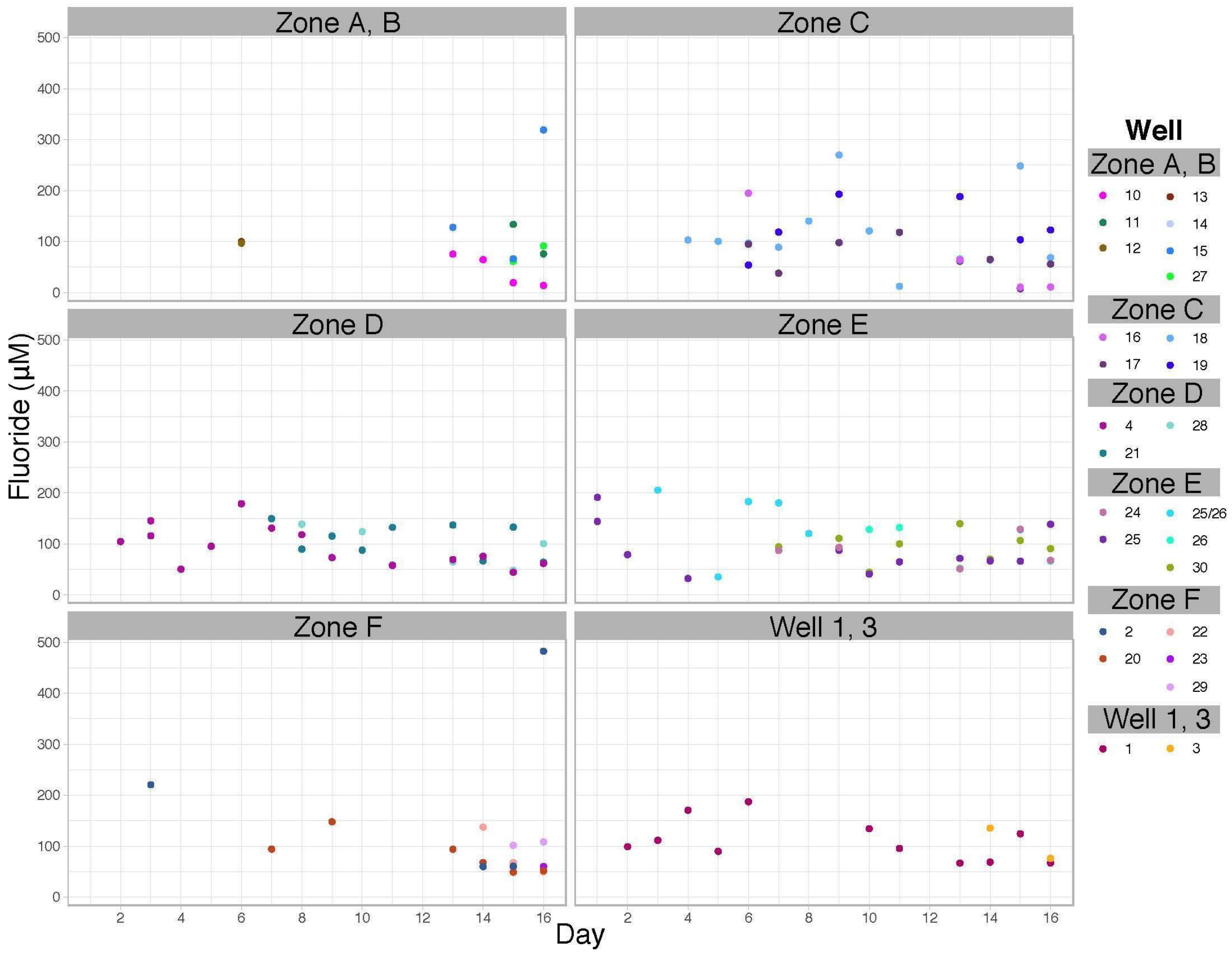

Zone A, B
Zone C
Zone D
Zone E
Zone F
Well 1, 3
Fluoride (µM)
Day
500
400
300
200
100
0
2
4
6
8
10
12
14
16
Well
Zone A, B
10
13
11
14
12
15
27
Zone C
16
18
17
19
Zone D
4
28
21
Zone E
24
25/26
25
26
30
Zone F
2
22
20
23
29
Well 1, 3
1
3

G.

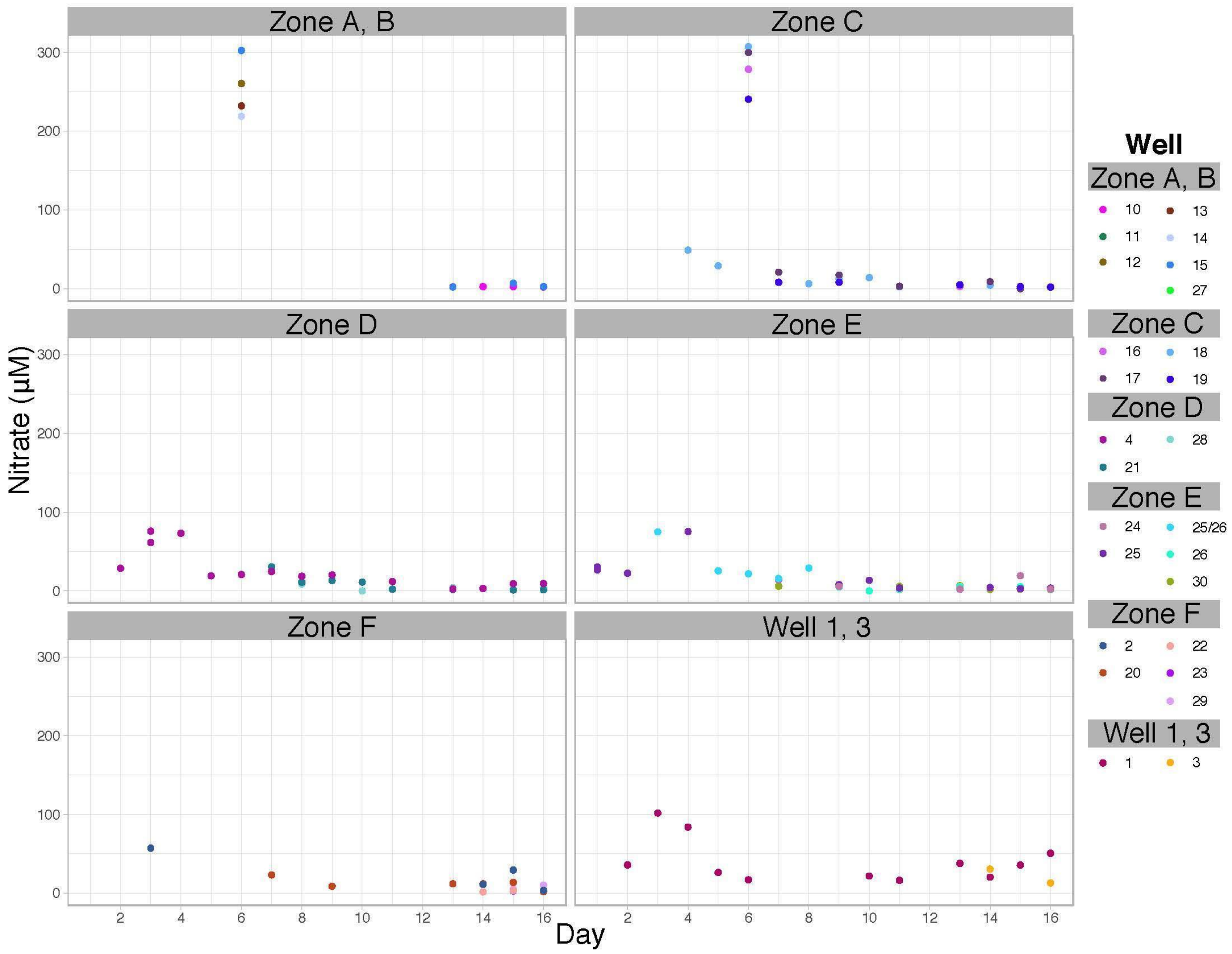

Zone A, B
Zone C
Zone D
Zone E
Zone F
Well 1, 3
Nitrate (µM)
Day
300
200
100
0
2
4
6
8
10
12
14
16
Well
Zone A, B
10
13
11
14
12
15
27
Zone C
16
18
17
19
Zone D
4
28
21
Zone E
24
25/26
25
26
30
Zone F
2
22
20
23
29
Well 1, 3
1
3

H.

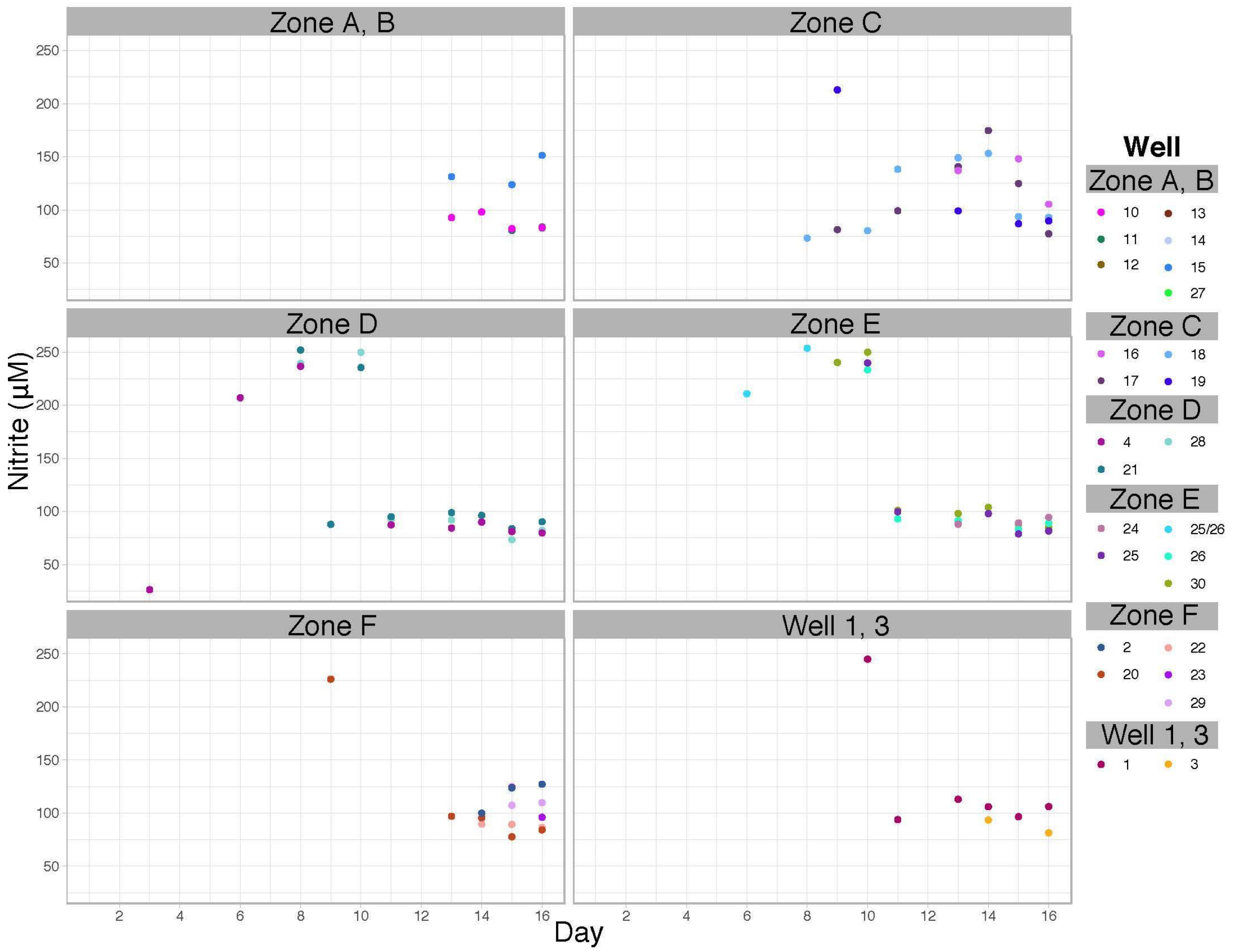

Zone A, B
Zone C
Zone D
Zone E
Zone F
Well 1, 3
Nitrite (µM)
Day
250
200
150
100
50
2
4
6
8
10
12
14
16
Well
Zone A, B
10
13
11
14
12
15
27
Zone C
16
18
17
19
Zone D
4
28
21
Zone E
24
25/26
25
26
30
Zone F
2
22
20
23
29
Well 1, 3
1
3

I.

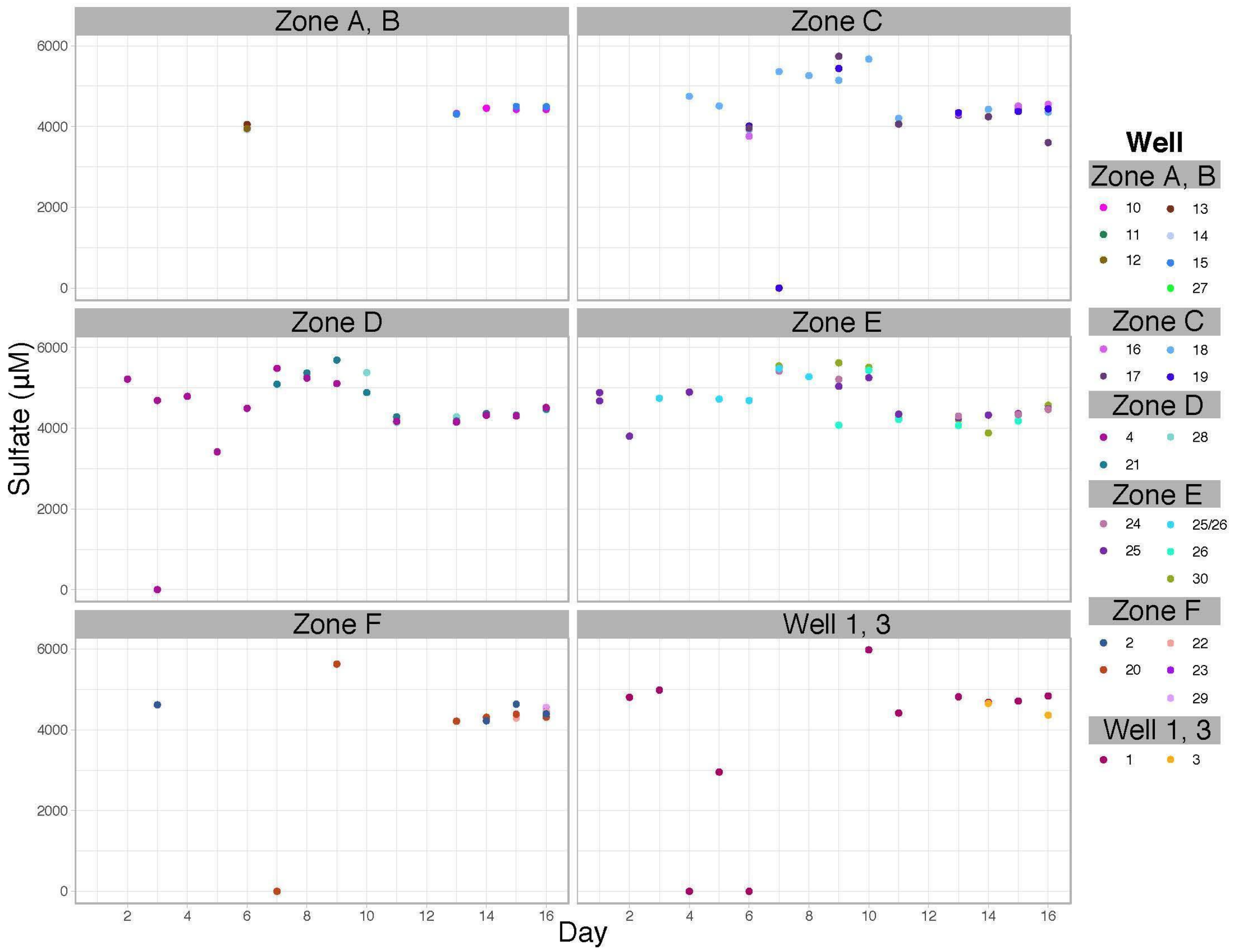

J.

Figure S7 A - J. Geochemical measurements in Plot B. Geochemical species, pH, and conductivity for Rifle site plot B during the course of this study. Grey panels at the top of sections indicate each zone (of plot B). Values reported for ammonia are only accurate up to 5544 µM. Measurements below the limit of detection are recorded here as zero.

Supplemental Tables

Table S1 Injection Scheme and Weather Data.
T = thunderstorms. All weather data from Garfield County Regional, CO NOAA weather station KRIL (39.5279N 107.72W), September 2016.

date	time	temp.°F	precip. (in), sum	injectate (g)			vol. injected (L)	
				urea	molasses	KBr	per load	total
1-Sep	4:30 pm	72	0.02	0	47	145	360	360
2-Sep	5:17 pm	71	0.15	180	0	0	360	720
3-Sep	9:45 am	69	0.23	180	0	0	377	1097
4-Sep	9:30 am	70	0	180	0	0	360	1457
5-Sep	11:00 am	68	T	180	0	145	360	1817
6-Sep	12:15 pm	65	0	0	47	145	360	2177
7-Sep		65	0	0	0	0	0	2177
8-Sep	11:20 am	65	0	0	398	145	360	2537
	3:00 pm			0	400	0	360	2897
9-Sep	10:20 am	64	0	1400	0	0	360	3257
	2:30 pm			1400	0	0	360	3617
10-Sep	9:30 am	62	0	1400	0	435	360	3977
	11:47 am			1400	0	0	360	4337
11-Sep	8:40 am	65	0	1400	0	0	360	4697
	11:30 am			1400	0	0	360	5057
12-Sep	10:45 am	67	T	1400	0	0	360	5417
	12:10 pm			1400	0	0	360	5777
13-Sep	9:40 am	64	T, 0.05	1400	0	0	360	6137
	12:45 pm			1400	0	0	360	6497
14-Sep	9:20 am	66	T, 0.03	1400	0	0	360	6857
	1:10 pm			1400	0	0	360	7217
15-Sep	9:10 am	58	0	1400	0	0	360	7577
	11:55 am			1400	0	0	360	7937
16-Sep		58	0	0	0	0	361	8298
TOTAL Injected:				20320 g	892 g	1015 g		8298 L

Table S2 Volume of groundwater filtered, ureolytic functional potential (copies of *ureC*), and alpha diversity for sediment and groundwater samples collected during the 16-day MICP study. NR = Not Recorded, GW = groundwater. Boxes marked in grey are below the detection limit (*E. coli K12* control) for copies of *ureC* in ground water samples.

sample name	well	time (day)	vol. filtered GW (mL); source	type	avg. *ureC* copies/µL	*ureC* per mL GW; or per g sediment	dsDNA (ng/µL)	Faith's PD
W4_1	4	1	600	water	12.5	0.02	0	88.58
W4_2	4	2	2000	water	3400	1.70	0.632	44.27
W4_3	4	3	500	water	4.7	0.01	0	43.36
W4_4	4	4	200	water	4.37	0.02	0	39.08
W4_5	4	5	NR	water	2500.67	-	2.35	31.59
W4_6	4	6	2100	water	1474.33	0.70	0.68	42
W4_8	4	8	275	water	6.23	0.02	0	51.18
W4_9	4	9	1200	water	1309	1.09	0.398	50.28
W4_10	4	10	1350	water	1242.67	0.92	3.18	68.94
W4_11	4	11	250	water	5.23	0.02	0	50.72
W4_13	4	13	100	water	4.8	0.05	0	61.75
W4_14	4	14	1900	water	5826.67	3.06	7.24	87.16
W4_15	4	15	1900	water	2076.67	1.09	4.22	49.72
W4_16	4	16	700	water	6.13	0.01	0	58.51
W1_1	1	1	2000	water	239	0.12	0.312	101.33
W1_16	1	16	1200	water	2453.67	2.04	28.2	83.02
W21_16	21	16	720	water	507	0.70	0.501	75.78
S4C_16	4C	16	ex-situ	sediment	2026.5	8106	25.2	52.11
S4_16	4	16	in-situ	sediment	13.23	52.92	20.6	76.54
S1_16	1	16	in-situ	sediment	12.97	51.88	23.4	69.04

S2_16	2	16	in-situ	sediment	37.83	151.32	15.3	62.53
S0_0	-	-	Colorado riverbank	environ. control	217.67	870.68	22.4	139.36
S4_0	0	0	inoculated	- control	7.7	30.8	21.6	133.13
-	-	-	uninoculated	- control	0.46	-	1.93	-
-	-	-	PowerSoil	- control	2.6	-	2.08	-
-	-	-	*S. pasteurii*	+ *ureC* control	2196	-	0.802	-
-	-	-	*E.coli K12*	- *ureC* control	36.4	-	4.06	-

Table S3. Rifle site background geochemistry, compared to this study. From the 1990, and 1999 DOE reports, we converted all background geochemical measurements to µM. Well 588 was adjacent to Plot B.

	**this study**	**1990, DOE**	**1999, DOE**
**Chemical Species (µM)**	**plot B**	**well 588**	**site average**
Ammonia (NH_3)	0 - 5544	38	7 - 17
Iron (Fe^{2+})	0 - 70	0 - 2	0 - 3
Sulfate (SO_4^{2-})	0 - 6000	6257 - 8328	351 - 14054
Nitrite (NO_2^-)	0 - 150	0 - 2	0
Nitrate (NO_3^-)	0 - 300	2 - 65	1 - 11
Chloride (Cl^-)	1250 - 3000	677 - 2003	3413 - 24116
Fluoride (F^-)	50 - 325	55 - 73	12 - 211
Specific Conductance, (µS/cm)	1500 - 3000	1190 - 1250	2170 - 3780
pH	6.8 - 8.3	6.7 - 7.01	7.04 - 8.54
